# Cellular frustration algorithms for anomaly detection applications

**DOI:** 10.1371/journal.pone.0218930

**Published:** 2019-07-08

**Authors:** Bruno Faria, Fernao Vistulo de Abreu

**Affiliations:** 1 Department of Physics, University of Aveiro, Aveiro, Portugal; 2 I3N Institute for Nanostructures, Nanomodelling and Nanofabrication, Aveiro, Portugal; Sichuan University, CHINA

## Abstract

Cellular frustrated models have been developed to describe how the adaptive immune system works. They are composed by independent agents that continuously pair and unpair depending on the information that one sub-set of these agents display. The emergent dynamics is sensitive to changes in the displayed information and can be used to detect anomalies, which can be important to accomplish the immune system main function of protecting the host. Therefore, it has been hypothesized that these models could be adequate to model the immune system activation. Likewise it has been hypothesized that these models could provide inspiration to develop new artificial intelligence algorithms for data mining applications. However, computational algorithms do not need to follow strictly the immunological reality. Here, we investigate efficient implementation strategies of these immune inspired ideas for anomaly detection applications and use real data to compare the performance of cellular frustration algorithms with standard implementations of one-class support vector machines and deep autoencoders. Our results demonstrate that more efficient implementations of cellular frustration algorithms are possible and also that cellular frustration algorithms can be advantageous for semi-supervised anomaly detection applications given their robustness and accuracy.

## Introduction

Cellular frustrated systems (CFSs) were originally developed to model the adaptive immune system [1–3]. A crucial hypothesis in these works was that the immune system should be extremely competent at detecting deviations from its normal functioning, i.e., at performing anomaly detection. This hypothesis guided the search for the simplest model that, on one side, would be compatible with experimental observations in immunology and, on the other, could perform these immune functions.

CFSs have the merit of making assumptions that are reasonable from an immune system perspective. However, from a computational point of view this is not necessarily an advantage. Nature has certainly been capable of finding solutions for complex tasks through natural selection. However, these solutions need not be computationally efficient nor entirely focused in solving the task of interest to the computational scientist. Biological systems explored solutions that were accessible to the natural system and in agreement with the physical world constraints. However, biological systems have also to contend with a number of other challenges and hence had to find solutions that are also robust in face of these challenges. For instance, the immune system has to contend with cell number fluctuations, spatial constraints, or the available cellular interaction mechanisms.

In this paper we developed an efficient algorithm inspired in cellular frustrated systems. Instead of respecting the acceptable mechanisms from an immunological point of view, we relax constraints that can improve computational efficiency without compromising anomaly detection performance. To accomplish this, a new algorithm was developed with the important discrimination mechanisms in mind. As a result, the results reported here are important because they show that immunity can be thought in more general terms, not necessarily linked to the biological reality.

The other goal of this paper is to compare the performance of the cellular frustration algorithm with state of the art algorithms on real datasets for anomaly detection applications. One of the difficulties in these problems is to understand what defines the normal behaviour [4–7], given that little or even no information is available from the anomalous class. Algorithms always have to make some assumption on what makes the anomalous class different. For instance, in one-class support vector machines (one-SVMs) normal samples are assumed to be concentrated, whereas anomalies are not [8, 9]. Whether these assumptions are adequate or not depends on the datasets. Therefore one challenge is to understand when, and how often these methods fail [4, 10–12].

This paper is organized as follows. In the following section we describe the different types of anomaly detection techniques and their relation to the cellular frustration framework (CFF). Then, we describe how anomaly detection is achieved within the CFF and define a cellular frustration algorithm (CFA) for anomaly detection applications. This algorithm gives special attention to the training stage, proposing a strategy that accelerates convergence. To gain a deeper understanding of the advantages of the new algorithm, a theoretical analysis is presented afterwards showing that the new strategy converges faster than the immunological models proposed in [1–3]. Next, the new algorithm is tested with several datasets, and a comparison is drawn with, not only the immunologically plausible version, but also state of the art algorithms in the literature, namely, support vector machines (SVMs) and autoencoders. Our results show that the current training algorithm converges faster than the immunological version and achieves similar, if not, more accurate performances. Furthermore, when compared with SVMs or autoencoders, it achieves similar accuracies, with higher robustness, that is, it produces better results in a wider range of scenarios.

## Brief review of anomaly detection approaches

The anomaly detection topic has a considerable history, having been first addressed in statistics [13], and recently readdressed in the data mining field [5, 14]. Anomaly detection appears in the literature under several names, such as one-class learning, novelty detection, change detection, outlier detection or even failure detection. This shows the enormous relevance given to this topic by many different communities, each with different histories, techniques, terminologies and with different applications in mind.

Anomalies can be defined as rare instances generated by mechanisms that differ from those generating normal instances. The goal of anomaly detection techniques is to detect signatures of anomaly in feature values. In principle, these signatures are extremes of the feature values distributions. Indeed, if anomalous instances have only features with values frequently found in normal instances, then they are indistinguishable from normal instances.

Finding signatures of an anomaly can be extremely challenging in data mining, because often normal instances have many features and statistical distributions with heavy tails. As a result, anomalies are not straightforwardly detected by the presence of a single extreme values; rather it is the number of extreme values and how they appear combined that is crucial to detect anomalies. Furthermore, many times a data pre-processing stage is required to expose anomalous patterns with the highest accuracies. This happens when one wants to monitor motors and industrial processes [15, 16], to analyse human behaviour [17, 18], whole communities [19], to gain information from small datasets or address big data challenges [20], to protect single computers [21], computer networks [22], to make efficient learning algorithms [23] or to provide inspiration on how biological systems work [24, 25]. In this work, we will assume that all preprocessing stages have already been performed or are not required. Indeed, the implementation of preprocessing strategies is a task specific to each problem, while here we will concentrate in general anomaly detection techniques.

Today, there are several data mining techniques addressing anomaly detection. They are generally divided in supervised (also known as binary classification), semi-supervised or unsupervised depending on the training required. Supervised techniques require training data with instances from the two categories, *normal* and *abnormal*. Semi-supervised techniques require only knowledge of normal instances. Unsupervised techniques use the available data to discern which instances are more likely to be distinct from the majority, i.e., anomalies.

Regardless of these distinctions, all these techniques try to detect a deviation from normality. How the different techniques establish the normality concept depends on the data and on the assumptions. The assumptions—e.g., the metrics in some distance based techniques and the criteria to establish where normal data lies—have a major impact in unsupervised techniques determining what can be detected. In classification these assumptions do not play such a critical role since the classification model can be adapted to the training data by tuning parameters. This makes unsupervised techniques less accurate, but simultaneously easier to use. Indeed, in many cases labelling data in categories is impossible. In this respect, semi-supervised techniques are a good alternative, since in many cases anomalies are rare and consequently their impact in training is small.

A fundamental difference exists between unsupervised or semi-supervised anomaly detection techniques and binary classification. In the first case a predictive model establishes what is different relatively to what is *normal*. Typically, outliers are samples lying far (in terms of a distance or a score) from a large fraction of the data. Therefore, unsupervised or semi-supervised techniques are concerned with establishing the boundary within which most data samples lie. By contrast, classification techniques are concerned in defining the best model that is capable of distinguishing the two classes. In this case, model parameters (weights), used to measure how far samples are from each other, are tuned so that samples in different classes lie away from each other.

Supervised (binary classification) techniques use labelling information to guide data separation. This information can nevertheless be misleading in the case of imbalanced datasets [7, 26–29], that is, datasets having many more instances of the normal class than the anomalous class. This is the case of interest in anomaly detection applications. However, most classification algorithms assume approximately balanced class distributions [27, 28]. When applied on datasets with an under-represented class they tend to favour the most represented class [27, 30]. Furthermore, since the anomalous class is under-represented it is unlikely that it will feature all types of anomalies. As a result supervised techniques are inadequate to identify anomalies that have not been presented in the training dataset [26]. This is particularly relevant for intrusion detection applications as the attacker will always attempt to explore these vulnerabilities. For these reasons, semi-supervised techniques can be more suitable for anomaly detection tasks since they build a descriptive model solely using information from the most represented class.

In practice, most techniques can be adapted to explore the different types of available data. For instance, support vector machines (SVMs) were initially developed by Vapnik for classification purposes [31, 32]. However, the scope of application of SVMs has been extended to tackle semi-supervised [33, 34] and unsupervised anomaly detection [35]. Still, SVMs were naturally defined as a classification technique and consequently, extensions required additional assumptions [36]. By contrast, cellular frustration algorithms (CFA) use training data to establish indicators of the normal class and therefore CFAs are naturally defined as semi-supervised techniques.

## Brief introduction to the cellular frustration framework

The Cellular Frustration Framework is an agent based modelling approach that received inspiration from the stable marriage problem (SMP) introduced by Gale and Shapley [37]. In the SMP there are two types of agents, man and woman, and each agent has a preference list where an ordering of preferences for agents of the other type is listed. The aim is to marry men and women in a stable configuration, i.e., such that no man and woman in two distinct marriages prefer to be married with one another than with their current mates. This problem found applications in economy, since it could represent the labour market, with employers on one side and employees on the other. Both sides, gain by establishing stable matchings as they could waste their time otherwise.

For anomaly detection purposes the CFF proposes a different formulation of the problem. First the two agent types should have specific functions. One type of agents presents the information to be evaluated by agents of the other type, which should react accordingly. Therefore, agents displaying information present very diverse traits and consequently agents of the other type can also have very diverse preference lists.

The important difference between the SMP and the CFF is that instead of searching for stable configurations, the CFF proposes looking at the dynamical properties of the population while it attempts to reach the stable configuration. What should matter is how long marriages survive and how their duration changes when new information is presented. In particular, it is possible to define populations of interacting agents that never form long-lived matchings, despite the fact that all agents attempt to form stable pairs [2]. This is due to the presence of frustration, as illustrated in the following example.

**Fig 1:**
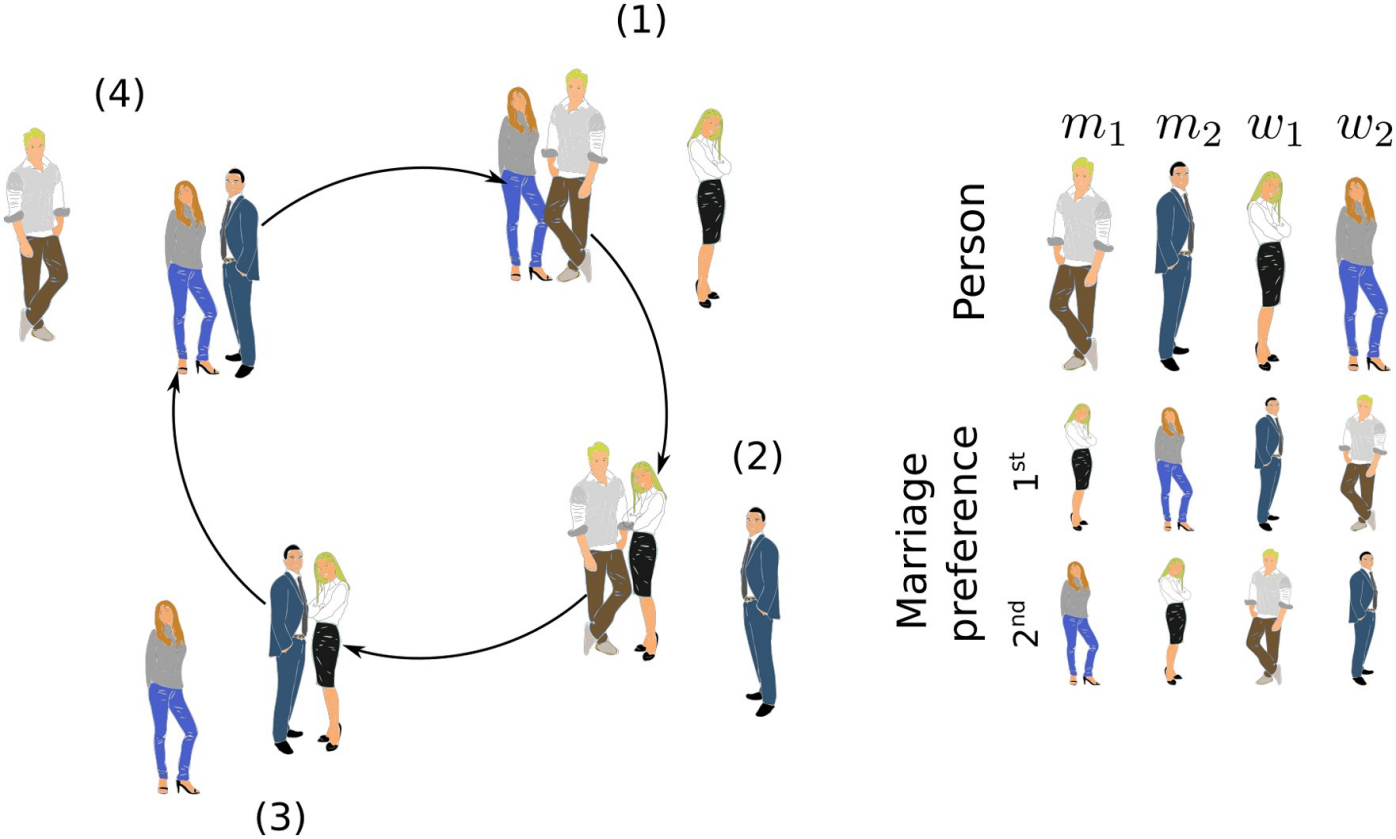
Illustration of the unstable dynamics generated by frustration in a population of many agents of two types and two sub-types of men and women. Men and women will always attempt to pair with individuals of the other type that maximize their satisfaction and which are ranked higher in the preferences lists on the right. It is supposed that men and women prefer to be paired than to be alone. It then follows from these preference lists that any pair can be destabilized by unmatched individuals in the population, because in any matching there is always one individual that is not completely satisfied. Cellular frustrated systems (CFSs) like this one are intrinsically unstable because even when all individuals are in stable pairs, breaking a small number of pairs is enough to destabilize the whole population and increase the number of unpaired individuals [2]. (Adapted from Faria BF, Mostardinha P, Vistulo de Abreu F (2017) Can the Immune System Perform a t-Test? PLoS ONE 12(1): e0169464. https://doi.org/10.1371/journal.pone.0169464.).

Consider a population with two types of men and women, pictured in Fig 1 by women (or men) dressed in casual or formal styles. Assume that men of type 1 (denoted *m*_1_) prefer women of type 1 (*w*_1_) to women of type 2 (*w*_2_); men of type 2 (*m*_2_) prefer women of type 2 (*w*_2_) to women of type 1 (*w*_1_), and so on as shown in the preference lists in Fig 1.

Assume that a man of type 1 marries a woman of type 2 (configuration (1) in Fig 1). Then, according to man’s preferences, if a woman of type 1 proposes to the man in the couple, he divorces and marries the proponent woman. However, next a man of type 2 can propose to the woman in the new couple, causing a divorce and forming another couple. The cycle can go on as illustrated in Fig 1, and it demonstrates the effect of frustration in the population: no agent can establish a stable pair because there will always be agents that can frustrate newly formed couples. This analysis can be made more general, to consider when all agents are different and that some are initially already paired. In any case, the main conclusion does not change: there are populations in which agents never form stable pairs [2].

Important consequences can result if major events in a population only take place when agents are matched for a minimum amount of time. This happens for certain reactions in biomolecular systems [38, 39], the cellular activation in immunology [1, 40, 41] or reproduction in evolutionary biology populations [42]). Then, it becomes clear that, despite the fact that all agents continuously interact, some will never react. This crucially depends on which agents are in the population and on the specific ordering of preferences.

Consider now that a third sub-type of women is introduced in the population. If one assumes that different men can have different preferences towards women they never saw, then approximately one third of the men population will rank women of the new type first. If all women of the third type have the same preferences towards men, then one sixth of them will establish stable marriages. This is a meaningful fraction which shows that: i) the highly frustrated dynamics require considerable organization on preferences orderings, and ii) the dynamics can be easily disrupted by foreign elements [2].

The cellular frustration framework used these ideas to propose an alternative view on how the human adaptive immune system is activated (i.e. triggered) [1, 41]. However, instead of men and women, there are two cell types: antigen presenting cells (APCs), and T cells. APCs present information to T cells through specialized ligands (formed by antigen bound to MHC molecules). T cells interact with these ligands with very different affinities. This information can be mapped onto a list (an interaction list or IList, similar to the preference list in the SMP) where ligands are ranked in order of decreasing affinities. T cells undergo a selection process, called the T cell repertoire education, which corresponds to a training stage. During this stage only normal (i.e., healthy) information is presented and only T cells engaging in a frustrated decision dynamics survive. Therefore, all T cells establishing long lived interactions are eliminated. This selection process establishes an ordering in T cells ILists.

**Fig 2:**
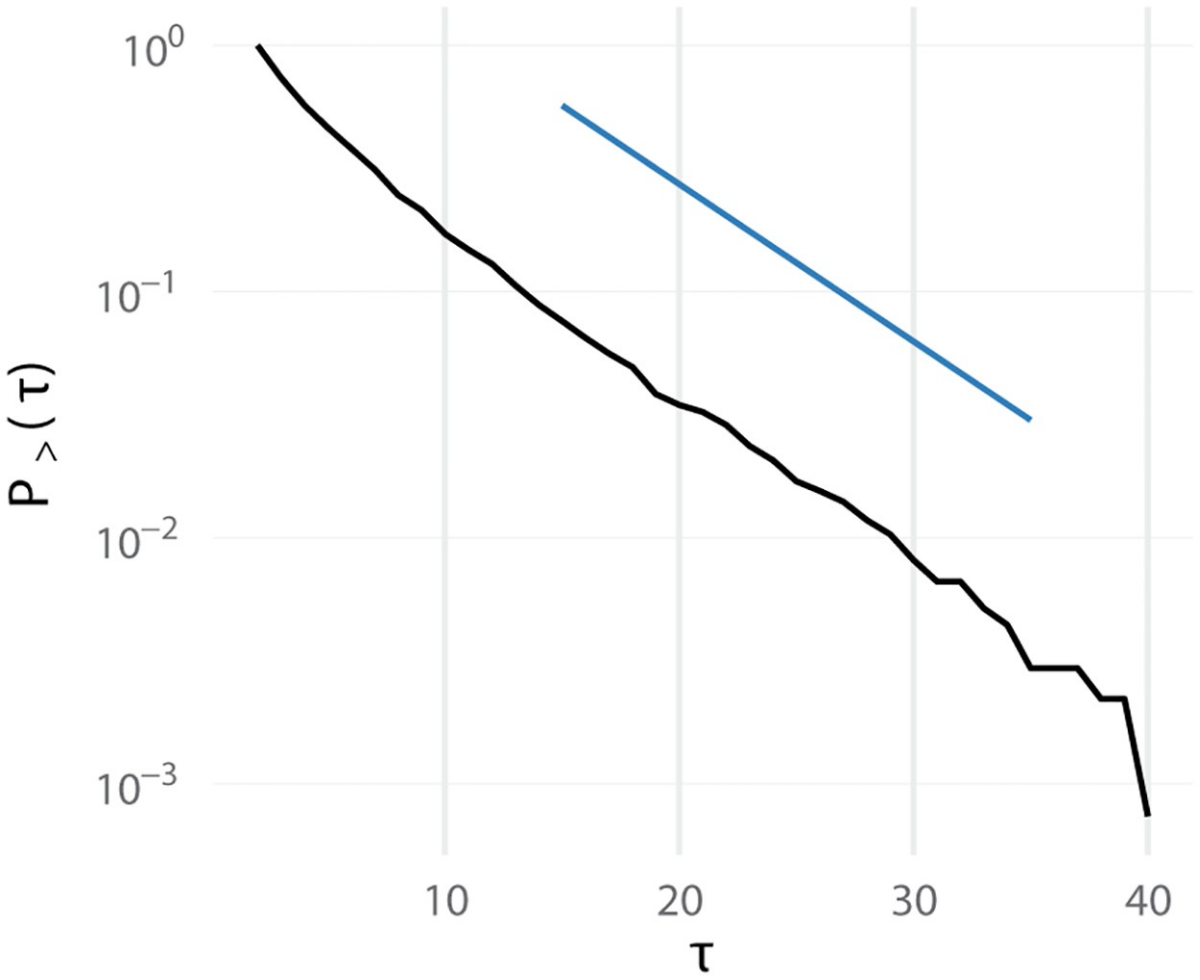
Typical curve for the frequency of contacts lasting more than *τ* iteration steps for a detector agent in a population engaging in a frustrated dynamics. For most agents in educated populations, the decay is approximately exponential as the one shown here. In this case a lifetime constant can be obtained from the slope of the depicted straight line. In non educated populations, the decay can be more complex, integrating the effect of processes with different lifetimes.

The frustrated decision dynamics whereby agents continuously pair and unpair can be characterized by a distribution of contacts with duration *τ*. For each agent, this distribution has an approximate exponential decay, with a characteristic decay constant defining the agent’s pairing lifetime (see Fig 2).

An important hypothesis used by the CFF is that pairing lifetimes are robust anomaly detection indicators. However, since accessing directly pairing lifetimes is difficult, the CFF proposes measuring the fraction of pairs lasting for a certain amount of time, *τ*, which is an indirect measure of the pairing lifetime. Indeed, it was hypothesized that the important function of positive selection—a training stage in the adaptive immune system that eliminates T cells that stay alone for too long—is to normalize the distribution of pairing durations so that by measuring a pairing duration, pairing lifetimes can be implicitly measured [3, 43].

Since all these concepts fit consistently in a common way of thinking of cellular populations, this was coined as the cellular frustration framework. In the next section we detail cellular frustration algorithms for data mining applications.

## Materials and methods

The cellular frustration framework was created to model the immune system. As a result, most assumptions related to the behaviour of cells were inspired in the current knowledge in immunology. Even though the immune system may have evolved to perform anomaly detection accurately, it had to withstand a number of challenges and constraints that algorithms for data mining applications do not need to be concerned with. Indeed, the immune system adopted the best solutions offered by chance and natural selection, and not necessarily the best solutions that can exist. In this section we describe improvements on the application of the cellular frustration framework for anomaly detection in data mining applications. We start by defining each agent and afterwards we describe how agents interact in the several stages of the algorithm. First we describe the education stage—commonly known as training in the anomaly detection field—and discuss how it can be optimized. Afterwards we describe the detection stage—also known as testing—and discuss how the performance of the algorithm is evaluated.

### Agents information and decision rules

**Fig 3:**
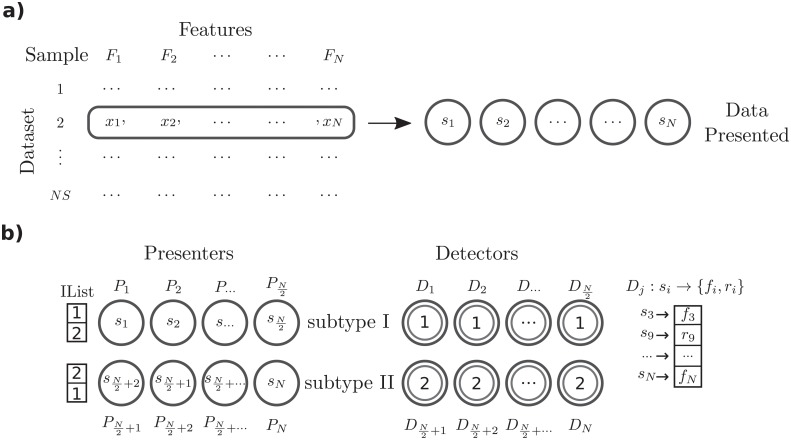
Illustration of the mapping of the information contained in a sample onto the cellular frustrated system. a) Transformation of sample values *x*_*i*_ from *N* features, *F*_*i*_, onto *non-overlapping* signals *s*_*i*_. b) Representation of the set of *N* agents in the model. The model is composed of two types of agents, presenters *P*_*i*_, and detectors *D*_*i*_, with two subtypes each, with an equal number of agents. Detectors either display 1 or 2 to presenters. Presenters either prioritize interactions with detectors displaying 1 or 2. This defines the two interaction lists (ILists) shown on the left and two subtypes of presenters. Detectors, on the other hand, perceive a wider range of signals. From interactions with each presenter, detectors either perceive a *f*_*i*_ or *r*_*i*_ signal which derive from signals *s*_*i*_ displayed by presenter agents. Most frequently *s*_*i*_ signals are mapped onto a *f*_*i*_ signal, and only rarely onto a *r*_*i*_ signal. This mapping varies from detector to detector as described in the text. Each detector has associated ILists and will prioritize establishing pairings with agents delivering signals that ranked in the highest positions. The IList for a detector *D*_*j*_ is shown on the right.

In the cellular frustration model considered here there are two types of agents (Fig 3). On one side there are *N* presenters *P*_*i*_ (the APCs in the immune system; *i* = 1, …, *N*) and on the other side, *N* detectors *D*_*i*_ (the T cells).

All agents are assigned interaction lists (ILists) where the information displayed by agents of the other type is ranked. These lists play the same role as the preference lists in the SMP. All agents change pair if the information displayed by an agent of the other type is ranked higher in their ILists than the information displayed by the agent they are paired with. Furthermore, as in the SMP, all agents prefer to be paired than to be alone. Computationally, decision rules and pair formation can be written as in the Pseudo-code 1.

**Pseudo-code 1** Function establishing pairing decisions when agents *a*_*i*_ and *a*_*j*_, of opposite types, are put in interaction. Both agents evaluate the ranking of the signals delivered by the other agent in the pair. Here $rank_{L_{i}}(s_{j})$ denotes the rank of signal *s*_*j*_ in agent’s *a*_*i*_ IList. {*s*_*i*_} is the set of signals displayed in a sample.

**function** Decision({*a*_*n*_}, *i*, *j*, {*s*_*i*_})

 **if** *a*_*i*_ is alone ∧ *a*_*j*_ is alone **then**

  pair *a*_*i*_ and *a*_*j*_

 **else if** *a*_*i*_ paired with *a*_*k*_ ∧ *a*_*j*_ is alone ∧

   $rank_{L_{i}}(s_{j})<rank_{L_{i}}(s_{k})$ **then**

  set *τ*_*i*_ and *τ*_*k*_ to 0

  unpair *a*_*i*_ and *a*_*k*_ from their current pairings

  pair *a*_*i*_ and *a*_*j*_

 **else if** *a*_*j*_ paired with *a*_*k*_ ∧ *a*_*i*_ is alone ∧

   $rank_{L_{j}}(s_{i})<rank_{L_{j}}(s_{k})$ **then**

  set *τ*_*j*_ and *τ*_*k*_ to zero

  unpair *a*_*j*_ and *a*_*k*_ from their current pairings

  pair *a*_*i*_ and *a*_*j*_

 **else if** *a*_*i*_ paired with *a*_*k*_ ∧ *a*_*j*_ paired with *a*_*p*_ ∧

   $rank_{L_{i}}(s_{j})<rank_{L_{i}}(s_{k})$ ∧ $rank_{L_{j}}(s_{i})<rank_{L_{j}}(s_{p})$ **then**

  unpair *a*_*k*_ and *a*_*p*_ from their current pairings

  pair *a*_*i*_ and *a*_*j*_

  set *τ*_*i*_, *τ*_*j*_, *τ*_*k*_ and *τ*_*p*_ to zero

 **end if**

**end function**

Interactions among agents in the population can be restricted by establishing that detectors can only interact with *C* presenters. This introduces the notion of connectivity in the model and in the examples presented below, the connectivity matrix is established by randomly drawing *C* presenters to each detector in the beginning of the simulation.

Following our previous work [43], it will be assumed that each agent can only perceive a binary signal, *b*, from the information displayed by agents of the opposite type. This simplifies considerably ILists and their orderings.

This simplification is extreme in the case of presenters ILists. In fact, in this model it is assumed that detectors present only two digits, 1 or 2. As a result, only two types of presenters ILists exist. This naturally organizes presenters in two subtypes (or groups), *I* and *II*, depending on whether they rank first the 1 or 2 digit, respectively (see Fig 3).

By contrast, detectors have access to a much more diverse information. This happens for two reasons. Firstly, because signals displayed by presenters arise from sample values, which can even be continuous variables. Secondly, because different presenters display information arising from different features.

We mapped the sample information in the *i*^*th*^ feature, *x*_*i*_, onto the binary signal perceived by a detector, *b*_*i*_, using two steps. First the *x*_*i*_ is mapped onto a signal *s*_*i*_ displayed by the *i*^*th*^ presenter, taking into account that all presenters present distinct (disjoint) information:

$$\begin{array}{c} s_{i}=i+\left(x_{i}-x_{i,min}\right)/\left(x_{i,max}-x_{i,min}+\epsilon \right) \end{array}$$ (1)

where *x*_*i*,*min*_ and *x*_*i*,*max*_ are the minimum and maximum in the whole dataset for the *i*^*th*^ feature, and *ϵ* is a small number (e.g., the machine epsilon number) needed to guarantee that different presenters display distinct information, i.e., {*s*_*i*_} ∩ {*s*_*i*+1_} = ∅, ∀*i*.

For each detector in the connectivity range of the *i*^*th*^ presenter, the signal *s*_*i*_ is mapped in a binary signal denoted by *f*_*i*_ or *r*_*i*_. This second mapping is such that, during training, *r* signals are rarely displayed, while *f* signals appear frequently. Therefore, the configurations perceived by detectors during training have mostly *f* signals.

**Fig 4:**
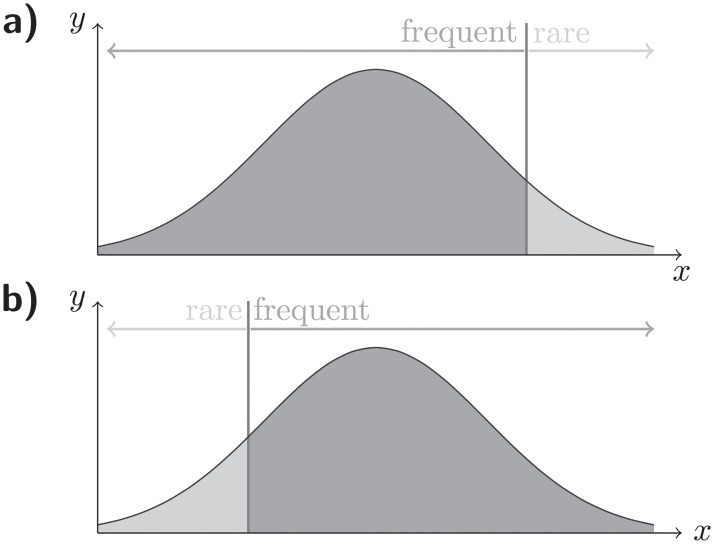
Mapping of a feature variable into frequent and rare signals. In this work detectors establish intervals mapping sample values onto rare signals either on the a) right, or on the b) left tails of the distribution function for each feature variable. The size of these intervals in the tails depends on the detector agent, and corresponds to *v*_*i*_% of the events observed during training. When *v*_*i*_ = 0%, then rare signals are not displayed during the training stage. In immunology these signals are called nonself ligands and in statistics they correspond to outliers.

Several different strategies could be used to define how each detector maps sample information onto rare and frequent signals. Here, we considered that for each feature *i*, a cumulative distribution function *F*_*i*_(*s*) that can be estimated from the data available for training. Then, detectors sense as rare signals, either values on the left or on the right tail of the associated distribution function (see Fig 4), i.e., for which *F*_*i*_(*s*_*i*_) < *v*_*i*_, or *F*_*i*_(*s*_*i*_) > 1 − *v*_*i*_, respectively. All other values are mapped onto frequent signals. The threshold probability *v*_*i*_, is different for each detector and is drawn from a uniform distribution between 0 and *v*_*max*_. Typically, *v*_*max*_ < 0.2 (i.e., 20%).

Note that, as mentioned before, the way detectors map the information displayed by different detectors has an impact on the detection accuracies achieved. For instance, the detectors considered here are one-sided, since only elements on one side of the distribution tail are mapped onto rare signals. Two-sided detectors could have also been considered but we leave these and other extensions for discussion in a forthcoming publication.

To summarize, cellular frustrated algorithms use two types of agents, presenters and detectors. All agents display information that is perceived, by agents of the opposite type, as binary signals. All presenter agents display different information, which derives from feature values, from a sample in a dataset. All agents pair and unpair continuously, favouring being paired with agents displaying information that is ranked in the highest positions in their interaction lists (ILists). I.e., agents pair and unpair as having preferences, in the same way as men and women try to match with the partners they prefer. During training, the information displayed by presenters can change from time to time. This changes the ranking of the perceived signals and has important implications in the pairing dynamics, as it will be discussed next.

### Training: Main concepts

To achieve accurate anomaly detection, cellular frustrated systems (CFSs) must first undergo a training stage (also called repertoire education) during which detector ILists are changed to increasingly frustrate the overall dynamics and reach a maximally frustrated state. To understand how this guarantees accurate anomaly detection, it is important to take into consideration the mechanisms involved, thoroughly discussed in [3] and [43]. So far it has been found that CFSs can detect 3 types of anomalous patterns: 1) the presence of outliers, i.e., signals never (or rarely) displayed during training; 2) the absence of an abnormally large number of frequently displayed signals (as compared to what is observed during training); 3) the absence of combinations of signals frequently displayed during training.

**Fig 5:**
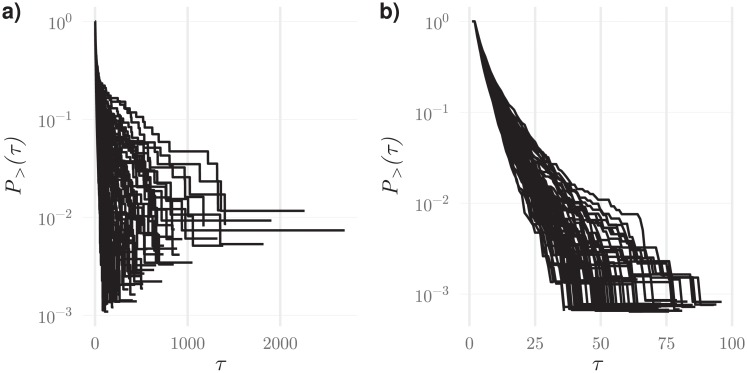
Typical plots of the frequency of contacts lasting longer than a time *τ* for all detector agents in a population, (a) at the beginning of the training stage and (b) in the end. At the beginning of training, some agents establish extremely long contacts. In the end of the process, all agents have similar dynamical behaviour with well defined pairing lifetimes.

**Fig 6:**
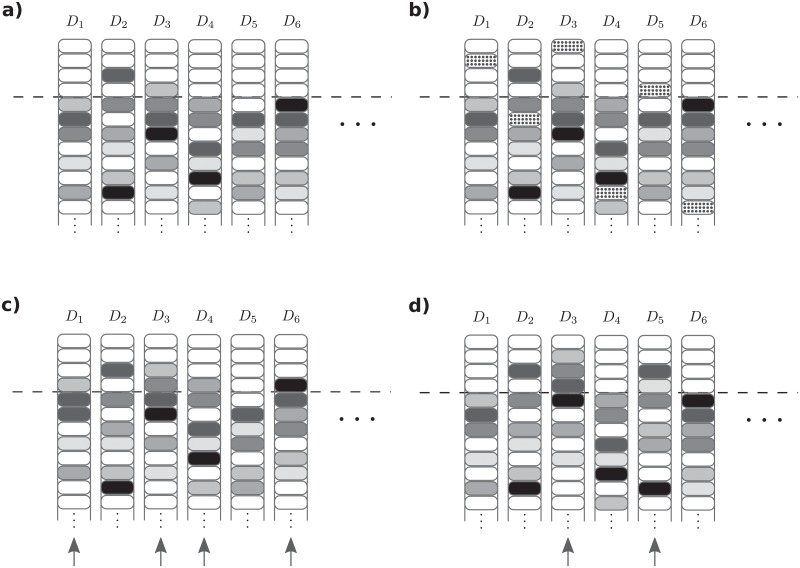
Schematic representation of the ordering in interaction lists (ILists) and their modification in the presence of anomalies. ILists for six detectors of the same subtype are represented schematically. White boxes represent signals delivered by presenters of the opposite subtype, and boxes in different grey shades represent signals delivered by different presenters of the same subtype of the detector. Only signals displayed by presenters for a given sample are represented; all others play no role in the dynamics and are omitted. a) ILists ordering when a normal sample is presented. Most detectors have on top positions—delimited by the dashed line—only signals delivered by agents of the opposite subtype. b) When a rare signal (represented in dotted boxes) that was not presented during training appears, detectors rank it in arbitrary positions; detectors *D*_1_, *D*_3_ and *D*_5_ can establish long lived interactions. c) When the number of frequently displayed signals going absent increases beyond what is typical during training, then several detectors can have less signals delivered by presenters of the opposite subtype on top positions (detectors *D*_1_,*D*_3_,*D*_4_ and *D*_6_), which can shift the remaining signals upwards mildly but on a large number of detectors. d) Even if the number of absent frequent signals is not larger than experienced during training, detection can be triggered if the absent signals were never absent together. In that case, shifts upwards in ILists can be stronger, although may affect a smaller number of ILists (detectors *D*_3_ and *D*_5_).

Detection of these three types of anomalies rely on the organisation of ILists during training. The goal of training is to maximize frustration homogeneously by reducing pairing lifetimes for all detectors in the population and across several samples (see Fig 5). This is accomplished by changing ILists of detectors paired for a time *τ* longer than a progressively reduced threshold pairing duration. To avoid establishing long-lived pairings, detectors should not rank on ILists top positions signals delivered by presenters of the same subtype. Instead, on top positions there should be a set of signals frequently displayed by presenters of the opposite subtype which can destabilize matchings with presenters of the same subtype. Therefore, after training the organisation of IList when normal samples are displayed, should be as represented in Fig 6a), with most detectors ILists having only signals displayed by presenters of the opposite subtype on the top. Note that in this figure, only signals displayed by presenters in the system are represented, since signals not displayed, play no role in the dynamics.

When anomalous samples are presented, detection occurs if signals delivered by presenters of the same subtype of the detector are ranked in higher positions, producing pairings with large durations *τ*. This can happen either because signals have not been presented during training, in which case they will be ranked in any position in ILists (Fig 6b), or because frequently displayed signals become absent and detectors ranking them on top positions will push the remaining signals upwards (Fig 6c and 6d). In the last case this can happen when a number of frequently displayed signals become absent in larger numbers than happened during training. This can have a mild impact in many detectors (Fig 6c). The other possibility is that combinations of signals frequently displayed together, become absent. This can have a stronger impact although in a smaller number of detectors (Fig 6d). In practice, all three mechanisms can operate simultaneously.

### Training: Algorithms

The detection mechanisms discussed above require an algorithm for ordering ILists. In [3, 43] it was proposed that the education of cells in the immune system is accomplished through a negative selection mechanism operating on the duration of pairings. Following inspiration from what is known in immunology, it was proposed that each T cell (or detector agent) establishing one of the longest pairings would be replaced by a new incoming cell, with randomly ordered IList.

Here we will show that this process can be speeded up considerably. Indeed, the immune system has a rather inefficient process of educating cells, which amounts to eliminate approximately 95% of thymocytes and replace them with new cells (with untested receptors). However, this inefficient process may be due to the fact that the immune system did not have access to mechanisms allowing edition of receptors. If one takes an artificial intelligence perspective, it is more reasonable to correct progressively ILists that led to stable pairings, instead of simply replacing them by new randomly ordered ILists. This can have the advantage of avoiding that new information destroys past experience. However, it requires designing a new strategy to order ILists.

In this article we discuss in detail a new and simple strategy. It consists in exchanging the signal that led to the longest pairings, with a randomly drawn signal from a lower position in the IList. This strategy pushes to lower positions signals delivered by presenters of the same subtype since they produce the longest pairings. Furthermore, it can bring to top positions signals that have never (or rarely) been displayed by presenters of the same agent subtype. Indeed, it should be noted that the signal randomly drawn from a position below, is not necessarily displayed in the current sample. As a result the strategy for correcting ILists can make detection of outliers more robust than the immunological plausible strategy of replacing a detector by a new detector.

The training algorithm can then be summarized as follows (see Pseudo-code 2). First, detectors ILists are initialized (line 2), being assigned a set of *C* randomly drawn presenters for interaction with each detector (*C* stands for the detectors connectivity).

**Pseudo-code 2** Repertoire training in CFAs

1: **function** Training(*t*_*max*_, *W*_*τ*_)

2:  Initialize *D*_*i*_ with a random IList with *f* and *r*

3:   signals from *C* randomly drawn presenters

4:  Initialize {*τ*_*i*_} to zero

5:  Initialize *τ*_*n*_ to *W*_*τ*_

6:  **for** *t* in 1 to *t*_*max*_ **do**

7:    Initialize *N*_*subs*_ to zero

8:    Initialize $\tau_{n}^{W}$ to zero

9:    **for** *t*_*w*_ in 1 to *W*_*τ*_ **do**

10:     **if** *t*_*w*_ (mod *T*_*S*_) is zero **then**

11:      change sample {*s*_*i*_}

12:     **end if**

13:     **for all** *a*_*i*_ in {*P*_*i*_} ∪ {*D*_*i*_} **do**

14:      *a*_*j*_: agent randomly selected from *a*_*i*_

15:       connectivity

16:      Decision({*a*_*n*_}, *i*, *j*, {*s*_*i*_})

17:     **end for**

18:     **for all** *a*_*j*_ in {*D*_*i*_} **do**

19:      **if** *τ*_*j*_ ≥ *τ*_*n*_ **then**

20:       **if** IS (immunological strategy) **then**

21:        randomly permute *a*_*j*_ IList

22:       **end if** AIS **then**

23:        *a*_*k*_: agent paired with *a*_*j*_

24:        p← random integer larger than

25:         $rank_{L_{j}}(s_{k})$

26:        In *L*_*j*_ swap content ranked at

27:         $rank_{L_{j}}(s_{k})$ with content ranked at p

28:       **end if**

29:       unpair *a*_*j*_ and set *τ*_*j*_ to zero

30:       *N*_*subs*_ ← *N*_*subs*_ + 1

31:      **end if**

32:     **end for**

33:     $\tau_{n}^{W}\leftarrow max(\tau_{j},\tau_{n}^{W})$

34:     Increment *τ*_*j*_ for all pairings

35:    **end for**

36:    **if** *N*_*subs*_ is 0 **then**

37:     $\tau_{n}\leftarrow \tau_{n}^{W}$, if $\tau_{n}^{W}<\tau_{n}$

38:    **end if**

39:   **end for**

40:   **return** {*D*_*i*_}

41: **end function**

Then, the iterated frustrated dynamics is run. At each time step, a randomly drawn agent is put in interaction with an agent with signals in its IList. A new pair is formed whenever the two interacting agents prioritize this interaction (see Pseudo-code 1). In that case, if they were already conjugated, former pairs are terminated. The process is repeated (lines 13-17) until all agents were given a chance to chose an agent of the opposite type to interact with.

Then every detector *D*_*j*_ involved in a pairing lasting *τ*_*j*_ iterations with *τ*_*j*_ > *τ*_*n*_ undergo IList education (lines 20-31). In the Pseudo-code 2, the two training strategies are considered. The immunological plausible strategy (IS) simply replaces the IList by a new randomly drawn IList (line 21) while the swapping operation in the IList is considered for the artificial intelligence strategy (AIS: lines 23-27).

If after *W*_*τ*_ iterations (typically 10000 iterations) no agents exceeded *τ*_*n*_, then *τ*_*n*_ is updated to the largest pair duration in the last *W*_*τ*_ iterations (line 37). Also, every *T*_*S*_ iterations the sample displayed by presenters is changed (lines 10-12).

Training stops when *t*, the counter registering the number of iterations, exceeds the maximum number of iterations, *t*_*max*_ (condition in line 6, in Pseudo-code 2). Then, the set of ILists, {*D*_*j*_}, is registered and added to a repertoire with independently educated ILists. It should be mentioned that instead of terminating training if the predefined number of iterations *t*_*max*_ is reached, other stopping criteria could be used, like considering stopping training if *τ*_*n*_ reaches a pre-defined value.

The function in Pseudo-code 2 is then called again to educate another set of ILists, where the same connectivity is assigned to each detector. This process is repeated *N*_*pop*_ times, so that in the end a repertoire with *N*_*pop*_ sets of independently educated ILists is established.

Building a repertoire of independently educated populations of detectors can improve the algorithm performance in the presence of outliers, as previously noticed in [3]. This happens because the probability of having ILists ranking rare ligands on top positions, not presented during training and displayed by presenters of the same subtype, is increased.

As a side note we remark that in this work it was avoided that the two signals (frequent or rare) delivered by a presenter of the opposite agent subtype are both ranked on top positions. Indeed this would not favour detection since the absence of the frequent signal would be compensated by the presence of the rare signal. Therefore we forced rare signals delivered by agents of the opposite subtype to be ranked (and frozen) on bottom positions in ILists. This improvement in the algorithms did not change results qualitatively, and for a matter of simplification in the presentation it was omitted from the pseudo-codes.

### Detection algorithm

Testing the anomaly detection performance of the algorithm follows closely that outlined in [43]. First it undergoes a calibration stage, to extract typical properties from the frustrated dynamics. In this stage agents engage in a frustrated dynamics using the decision rules in the Pseudo-code 1. However, a process termed anergy is now introduced, terminating pairings lasting longer than *τ*_*A*_ and replacing the detector involved by another detector in the repertoire with the same connectivity (Pseudo-code 3). In our results we used *τ*_*A*_ = 5 iterations. During calibration only normal samples (from the normal dataset) available for training are used. The dynamics is run for *W*_*d*_ iterations for each sample (typically *W*_*d*_ = 10^4^ iterations). The number $c_{i,s}^{0}(\tau_{act})$ of long-lived pairings that lasted longer than *τ*_*act*_ iterations and involving a presenter with index *i* when sample *s* is presented is incremented. Defining the ordered vector $c_{i,(j)}^{0}(\tau_{act})$, such that $c_{i,(j)}^{0}(\tau_{act})\geq c_{i,(j+1)}^{0}(\tau_{act})\forall j$, then an activation threshold is established by defining $n_{i}^{0}(\tau_{act})=c_{i,(x)}^{0}(\tau_{act})$ where *x* = *N*_*c*_ × *f*, with *N*_*c*_ the number of samples used during the calibration and *f* is a real number between 0 and 1. Typically we use *f* = 0.1, and hence the 10% largest number of pairings lasting a time larger than *τ*_*act*_ in a sample are considered. The activation reference time was chosen to be equal to the largest pairing time during calibration, i.e., *τ*_*act*_ = *τ*_*A*_.

**Pseudo-code 3** Monitoring stage of the cellular frustration algorithm.

1: **function** Monitoring(*W*_*d*_, {*P*_*i*_}, {*D*_*i*_}, *τ*_*A*_, {*s*_*i*_})

2:  Initialize {*τ*_*i*_} to zero

3:  Initialize *c*_*i*,*s*_(*τ*) to zero

4:  **for** *t*_*w*_ in 1 to *W*_*d*_ **do**

5:   **for all** *a*_*i*_ in {*P*_*i*_} ∪ {*D*_*i*_} **do**

6:    *a*_*j*_: agent randomly selected from *a*_*i*_

7:    connectivity

8:    Decision({*a*_*n*_}, *i*, *j*, {*s*_*i*_})

9:   **end for**

10:   **for all** *a*_*j*_ in {*D*_*i*_} **do**

11:    **if** *τ*_*j*_ ≥ *τ*_*A*_ **then**

12:     Separate *a*_*j*_ from *a*_*k*_ and set *τ*_*j*_ and *τ*_*k*_

13:     to zero

14:     *c*_*i*,*s*_(*τ*_*A*_) ← *c*_*i*,*s*_(*τ*_*A*_) + 1

15:     *c*_*k*,*s*_(*τ*_*A*_) ← *c*_*k*,*s*_(*τ*_*A*_) + 1

16:     Replace *a*_*j*_ with a random detector

17:      with the same connectivity

18:    **end if**

19:   **end for**

20:   Increment all *τ*_*i*_

21:   Increment all *c*_*i*,*s*_(*τ*_*i*_)

22:  **end for**

23:  **return** {*c*_*i*,*s*_}

24: **end function**

To evaluate detection capabilities the decision dynamics is run in the testing stage in the same conditions as in the calibration stage. Presenters display either information from $N_{d}^{s}$ samples from a self-dataset, or $N_{d}^{ns}$ samples from a nonself or abnormal-self dataset. Several examples are illustrated in the Numerical Results section. The CFS response to the information displayed by sample *s* is calculated using the normalized number of pairings, ${\tilde{c}}_{i,s}(\tau_{act})=c_{i,s}(\tau_{act})/c_{i,s}(0)$, ${\tilde{n}}_{i}(\tau_{act})=n_{i}^{0}(\tau_{act})/n_{i}(0)$ according to:

$$\begin{array}{c} R_{s}=\sum_{i}\left({\tilde{c}}_{i,s}\left(\tau_{act}\right)-{\tilde{n}}_{i}^{0}\left(\tau_{act}\right)\right)\theta \left({\tilde{c}}_{i,s}\left(\tau_{act}\right)-{\tilde{n}}_{i}^{0}\left(\tau_{act}\right)\right) \end{array}$$ (2)

where *θ* is the Heaviside function. Thus the CFS response sums the increments on the number of long pairings relatively to the calibration stage, using the (normalized) number of pairings in the time interval *W*_*d*_.

To quantify the detection accuracy we compute the true positive rate for a fixed false positive rate, *FPR*. To achieve this we create and ordered vector of population responses to the $N_{d}^{s}$ normal samples displayed in the testing stage, $R_{\left(i\right)}^{s}$, such that $R_{\left(i\right)}^{s}\geq R_{\left(i+1\right)}^{s}\forall i$ and find $R_{x}^{s}$, where $x=N_{d}^{s}\times FPR$. Then the true positive rate becomes $TPR=\#\{R_{s}^{ns}:R_{s}^{ns}>R_{x}^{s}\}/N_{d}^{ns}$, where $R_{s}^{ns}$ are the population responses to the $N_{d}^{ns}$ samples displayed with anomalies. The true positive rate is thus equal to the fraction of samples displaying anomalies with responses greater than $R_{x}^{s}$.

## Results

### Training convergence: Theoretical results

In this section we will use a quantitative approach to understand how much faster the training strategy proposed above is, relatively to the immunologically more plausible alternative. This analysis has also the merit of highlighting a computational constraint arising on the ordering of interaction lists by education mechanisms. In the immunological plausible algorithm this is particularly striking since training has only an effect on a few top positions. Yet, the existence of a limited number of ordered positions is required to accomplish anomaly detection. In particular, if interaction lists were completely ordered, no anomaly detection would result [43]. In fact, the number of ordered positions is a function of the variability in the input data samples that characterize normal states. This is an emergent property of the population of agents selected after training. For this reason, modelling the ordering of interaction lists can be insightful and here we provide an initial approach to this issue.

Here we consider a simpler, yet similar task, capturing the essential differences between the two approaches but reducing the complexity of the problem to that of ordering a single IList.

The simpler model assumes that there are *N* items of two types (*N*/2 from each type) in a IList. By definition, it is assumed that one type of items is *correctly* ranked if they are ranked in top positions. Conversely, when items of the other type appear in top positions they are *incorrectly* ranked. The aim is to find how many iterations are necessary to obtain an IList with *n* correctly ranked items in the top *n* positions, using two different algorithms.

The first algorithm bears inspiration from the immunological negative selection model. On each time step an item is selected from the IList. If the item is incorrectly ranked in the top *n* positions, then a random permutation is operated on the whole IList, which corresponds to replacing the IList by a new one. This simulates the interaction of detectors with presenters producing long pairings and the subsequent negative selection of the detector.

The second algorithm reproduces the artificial intelligence training strategy, whereby selection of an incorrectly ranked item in the top *n* positions swaps the incorrectly ranked item with a randomly selected item from the *N* − *n* positions below.

**Fig 7:**
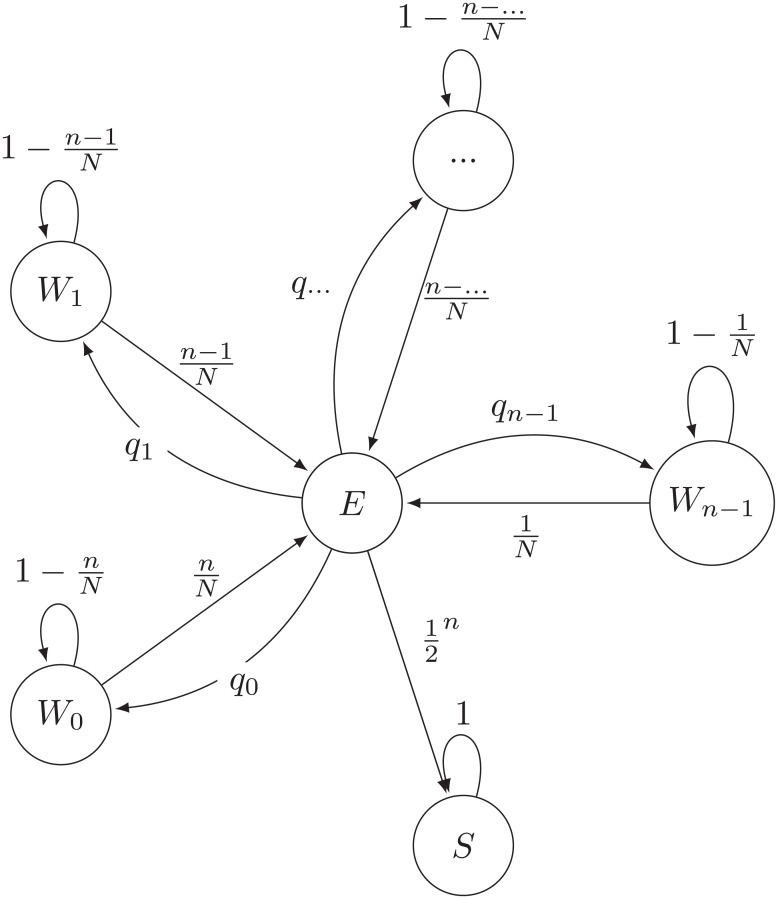
Markov chain describing the state transition that detectors undergo during training considering the immunological model. In this representation, *n* describes the number of top positions to be corrected in a list of size *N*, *W*_*m*_ the waiting states, that represent a list with *m* correctly ranked items on the top *n* positions and *E* the education state. The probability of transition from the list education state *E* to the waiting states, *W*_*m*_, is $q_{m}=\left(\begin{array}{c} \begin{array}{c} n\\ m \end{array} \end{array}\right){\left(1/2\right)}^{n-m}{\left(1/2\right)}^{m}$. The transition probability from a waiting state, *W*_*m*_, to the education state, *E*, is *q*_*educ*_ = (*n* − *m*)/*N*.

**Fig 8:**
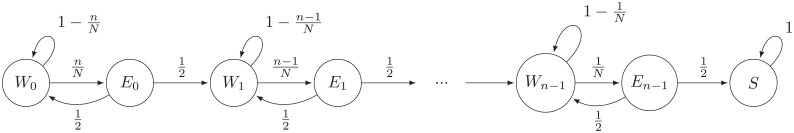
Markov chain describing the state transition that detectors undergo during training considering the new approach for *IList* correction. Here, *n* describes the number of top positions to be corrected in a list of size *N*, *W*_*m*_ the waiting states that represent a list with *m* correctly ranked items on the top *n* positions and *E*_*m*_ the different education states. The probability of transition from the waiting state *W*_*m*_ to the education state *E*_*m*_ is *q*_*educ*_ = (*n* − *m*)/*N*. However, contrarily to the immunological plausible strategy, lists are progressively corrected. Hence, once a given waiting state *W*_*m*_ is reached, the list can either go to a waiting state *W*_*m*+1_ or stay in the same waiting state. The probability of transition from the education state *E*_*m*_ to the waiting states *W*_*m*_ or *W*_*m*+1_ are approximately equal to (1/2), when *N* ≫ *n*.

The two algorithms can be modelled with the Markov models graphically represented in Figs 7 and 8. These models consist of waiting states with *m* correctly ranked items in the top *n* positions, *W*_*m*_, transient education states, *E* or *E*_*i*_, on which the two different training strategies operate, and the absorbing state *S* that stops the algorithm when all items are correctly ranked on the top positions.

A fundamental difference exists between the two models. In the immunological model IList education can send the model to a *W*_*m*_ state with any number *m* of correctly ranked items. These states have different probabilities of sending the system to the education state *E*, which depends on the number of incorrectly ranked items. When there are *m* correctly ranked items this probability is *q*_*educ*_ = (*n* − *m*)/*N*. If the list is sent to education, state *E*, the immunological model replaces the list by a new randomly drawn list. Therefore, from state *E* the system goes onto a state with *m* of correctly ranked items with probability $q_{m}=\left(\begin{array}{c} \begin{array}{c} n\\ m \end{array} \end{array}\right){\left(1/2\right)}^{n-m}{\left(1/2\right)}^{m}$. In particular, it reaches the absorbing state with probability 1/2^*n*^. Clearly, the larger *n* the harder it takes to completely order the top positions in the list.

By contrast, in the artificial intelligence approach lists are progressively corrected. The associated Markov model has a quite different diagram as shown in Fig 8. In fact, each time a list enters education, which happens with the same probability as before *q*_*educ*_ = (*n* − *m*)/*N*, when it has *m* correctly ranked items, then it either places a correctly ranked item in that position or not. Here we assume that the total number of items in the list is much larger than the number of positions to educate, *N* ≫ *n*, so that both these probabilities can be assumed to be equal to 1/2. As a result, in the artificial intelligence approach the system progresses along progressively more educated lists (states *W*_*m*_), although it only corrects one item at each time.

These two Markov models can be described by different transition matrices, containing the probabilities of transition, *p*_*ij*_, from a state *i* to a state *j*. In the case of the immunological plausible strategy, this is:

$$P_{IS}=\left(\begin{array}{cccccc} E & W_{0} & W_{1} & \cdots & W_{n-1} & S\\ 0 & (\begin{array}{c} n\\ 0 \end{array})\frac{1}{2^{n}} & (\begin{array}{c} n\\ 1 \end{array})\frac{1}{2^{n}} & \cdots & (\begin{array}{c} n\\ n-1 \end{array})\frac{1}{2^{n}} & \frac{1}{2^{n}}\\ \frac{n}{N} & 1-\frac{n}{N} & 0 & \cdots & 0 & 0\\ \vdots & \vdots & \vdots & \ddots & \vdots & \vdots \\ \frac{1}{N} & 0 & 0 & \cdots & 1-\frac{1}{N} & 0\\ 0 & 0 & 0 & \cdots & 0 & 1 \end{array}\right)$$ (3)

while for the case of the artificial intelligence strategy, it becomes:

$$P_{AIS}=\left(\begin{array}{cccccc} W_{0} & E_{0} & W_{1} & E_{1} & \cdots & S\\ 1-\frac{n}{N} & \frac{n}{N} & 0 & 0 & \cdots & 0\\ \frac{1}{2} & 0 & \frac{1}{2} & 0 & \cdots & 0\\ 0 & 0 & 1-\frac{n-1}{N} & \frac{n-1}{N} & \cdots & 0\\ 0 & 0 & \frac{1}{2} & 0 & \cdots & 0\\ \vdots & \vdots & \vdots & \vdots & \ddots & \vdots \\ 0 & 0 & 0 & 0 & \cdots & 1 \end{array}\right)$$ (4)

To calculate the average number of steps, *K*_*i*_, required to reach the absorbing state starting from state *i*, one considers an ensemble of lists starting in state *i*, and the ensemble of these lists in the following iteration. The average number of steps for these different configurations of lists to reach the absorbing state should differ by 1 iteration. Therefore we should have *K*_*i*_ = 1 + ∑_*j*_ *p*_*ij*_ *K*_*j*_, where the sum goes over all possible states and accounts for the average number of steps required to reach the final state starting from the following configuration.

Using this equation for the immunological plausible approach it can be noted that every state *W*_*m*_ can be written in terms of state *E* as:

$$\begin{array}{c} K_{W_{m}}=K_{E}+\frac{N}{n-m} \end{array}$$ (5)

Substituting Eq (5) in the equation for the *E* state, we arrive at an expected number of steps to absorption of:

KE=2n+N∑j=0n-1(nj)1(n-j),n>1 (6)

Using this solution in Eq (5) we get the expected number of steps to absorption from a waiting state *W*_*m*_:

Kwm=Nn-m+2n+N∑j=0n-1(nj)1(n-j),∀m<n,n>1 (7)

Writing a general expression for the expected number of steps to absorption using the artificial intelligence strategy requires noting two conditions. First, that the expressions for the *E*_*m*_ states can be written in terms of the expressions for the waiting states, hence:

$$\begin{array}{c} K_{E_{m}}=1+\frac{1}{2}K_{W_{m}}+\frac{1}{2}K_{W_{m+1}} \end{array}$$ (8)

Next, by using this expression in the expression for the waiting states a pattern emerges:

$$\begin{array}{c} K_{W_{m}}=\frac{2N}{n-m}+2+K_{W_{m+1}} \end{array}$$ (9)

Rewriting Eq (9) in terms of the absorbing *S* state gives:

$$\begin{array}{c} \begin{array}{c} K_{W_{m}}=2\left(n-m\right)+2N\sum_{j=m}^{n-1}\frac{1}{n-j},\\ \forall \;0\leq m\leq n-1,n\geq 1 \end{array} \end{array}$$ (10)

**Fig 9:**
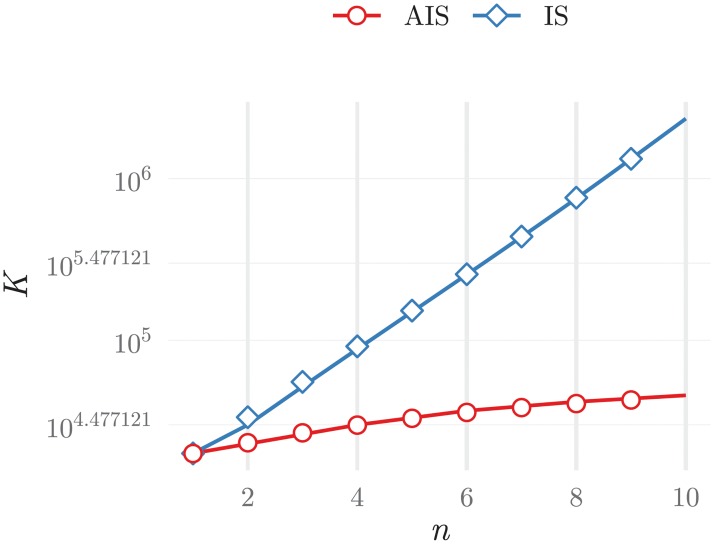
Average number of iterations required to find a list with all items in the top *n* positions correctly ranked, for the two strategies discussed in the text: The immunologically plausible strategy (IS) and the artificial intelligence strategy (AIS).

Expressions (7) and (10) allow comparing the convergence speed for the two strategies. In Fig 9, it can be appreciated that the two strategies have very different convergence speeds even when only a small number of items has to be correctly ranked. Importantly, this difference can be of an order of magnitude.

In the next section this result is tested with the education of all ILists in a population. A fundamental difference exists, which is that all ILists have to be educated simultaneously, interfering in the education of each other.

### Numerical results

Here, we will use numerical results to address the two issues discussed above, namely, on the speed of convergence and on the accuracy of the new training algorithm proposed here. For these tests four different datasets were used, three from the UCI repository [44] and one available at [45]. The datasets used concern: the evaluation of wine quality [46], the well known iris dataset for species discrimination using morphological measurements [47], discrimination of two types of surfaces using scattered sonar signals (the Connectionist Bench dataset [48]) and the identification of damaged or used ball bearings (ball bearings [45]).

These datasets have samples labelled in more than one class. Hence, they are most suited for supervised classification tasks. However, for the purpose of this paper we want to evaluate our algorithm in anomaly detection. This required defining which samples belong to the normal class, presenting a sub-set of them in a training stage and presenting the remaining samples in a testing stage. In some cases, in the original dataset the number of samples in one class was too small to obtain reliable results. In those cases groups of contiguous classes were created to define the normal and abnormal classes.

An important issue concerns the mapping of the information contained in samples with a very small number of features. When the number of agents is too small, the system could be blocked in a stable matchings configuration. Since our approach relies on the dynamical properties of the system, this should be avoided, which can be easily done by simply increasing the number of agents in the system. This was done by replicating an even number of times the population until reaching a number of presenters greater than 32. In the supplementary material S1 Fig we provide numerical simulations that show that for populations with more than 32 presenters the system does not get blocked in stable configurations.

For the two studies addressed in this work—on the computational performance and on the accuracy of the new algorithm—10 fold Monte-Carlo cross-validation was used. This amounted to randomly select 10 different normal datasets for training and testing, and running the algorithm under the same conditions.

To better establish the anomaly detection performance of cellular frustrated algorithms, we will also evaluate the performance of two state of the art type of algorithms in anomaly detection studies: support vector machines [49, 50] and autoencoders [51–55]. The strategy adopted was to use standard implementations of these methods, to evaluate the type of results non-experts would obtain if they used the available information in the literature. This point of view is tenable since, typically, in anomaly detection, one does not have access to additional information on the nature of anomalies.

For implementation of support vector machines it was used the well-known libsvm library [56], with a polynomial kernel with degree 2 and *c* = 0 and *v* = 1/*N*_*f*_. We noted that this kernel produced better results than the gaussian (*RBF*) kernel, which is used more often in classification problems. In the case of autoencoders, the *H*2*O* library [57] was used, with a network structure having 3 hidden layers (deep autoencoder) [51], where the inner layers have, respectively, *N*_*f*_ /2, *N*_*f*_ /4 and *N*_*f*_ /2 activation units. In all units, *tanh* activation functions were used. All remaining parameters were left to default values.

In the next subsection we describe the several datasets in greater detail. Afterwards we will use numerical results to discuss: the speed of convergence of the algorithm proposed here, the anomaly detection accuracy, its robustness and, finally, the mechanisms at play.

#### Datasets

Four datasets were used in the following studies. They are briefly denoted by ball bearings, iris, sonar and wines.

The ball bearings dataset [45] derives from Fast Fourier transforms (fft) of acceleration time series signals in essays with new or worn out (broken, damaged or even used) ball bearings. There are *N*_*f*_ = 32 features and 4150 samples deriving from essays with new ball bearings and 913 from worn out ball bearings. Training for anomaly detection tests used 500 samples from either, new or worn out ball bearings samples (Table 1).

**Table 1:** Number of examples from each category in each test for training and testing, for the different datasets used.

dataset	normal training data	number of set examples		
		train	test	
		normal	normal	abnormal
ball bearings	new	500	3650	913
	worn out	500	413	4150
iris	setosa	17	33	50
	versicolour	17	33	50
	virginica	17	33	50
sonar	metal	50	47	111
	rock	50	61	97
wines	3,4,5	500	1140	3258
	4,5,6	500	3318	1080
	5,6,7	500	4035	363
	6,7,8	500	2753	1645
	7,8,9	500	560	3838

The iris dataset was introduced by R. A. Fisher and is probably the most widely known dataset in the pattern recognition literature. This dataset comprises 50 samples describing three types of iris flowers by their width and length of petal and sepal (*N*_*f*_ = 4). Anomaly detection tests used a subset of samples from either one of the three classes for training, while examples from the other flower types were considered anomalous (Table 1).

The sonar dataset was collected by T. Sejnowski and R. Paul Gorman for discerning two types of surfaces using scattered sonar signals. The two surfaces considered were a roughly cylindrical rock and a metal cylinder. Several examples have been collected for the two surfaces at different angles and conditions. Overall signals have *N*_*f*_ = 60 features capturing information from reflected ultra-sounds and there are 97 samples from rock surfaces and 111 samples from metal surfaces. Again, tests considered that either type of material could work as the normal dataset.

Finally, in the wine dataset 4898 white wines are characterized in terms of *N*_*f*_ = 11 chemical-physico properties, such as pH, alcohol, fixed or volatile acidity, etc. A quality score from wine tasting evaluation is also provided. In practice scores from 3 to 9 have been awarded, 3 corresponding to a very bad wine, while 9 is awarded to wines of astounding quality. The aim of this dataset is to predict wine quality based only on physiochemical properties.

The number of wines scored with each score varies considerably. Wines evaluated with scores 3 and 9 are only a few: 20 and 5 respectively. Likewise, wines evaluated with scores 4 and 8 represent only a small fraction (∼3% each) of the total. Finally, wines evaluated with scores 5, 6 and 7 appear respectively 30%, 45% and 18% of the times.

To evaluate the anomaly detection algorithm it was necessary to define which sub-set of wines defined the normal class. To avoid having normal classes with too few examples, groups were defined with wines scoring 3,4 and 5, or 4,5 and 6, etc. (see Table 1). It was then possible to define sub-sets of 500 wines for training, and use the remaining for testing (Table 1).

#### Convergence tests

The first numerical results reported here concern the speed of convergence of the new AIS training algorithm as compared with the immunologically plausible strategy. In Table 2 the average number of iterations required to reduce all pairing durations below 180 iterations are shown.

**Table 2:** Average number of iterations required to reduce all pairing durations below 180 iterations during *W*_*τ*_ iterations (results in millions of iterations).

dataset	normal training data	training strategy	
		AIS	IS
ball bearings	new	0.5 ± 0.07	8 ± 5
	worn out	0.5 ± 0.1	6 ± 4
iris	setosa	0.5 ± 0.4	7 ± 4
	versicolour	0.5 ± 0.06	6 ± 4
	virginica	0.5 ± 0.1	7 ± 4
sonar	metal	1.3 ± 0.4	13 ± 5
	rock	1.4 ± 0.4	15 ± 4
wines	3,4,5	0.7 ± 0.2	11 ± 5
	4,5,6	0.6 ± 0.2	10 ± 5
	5,6,7	0.7 ± 0.3	11 ± 5
	6,7,8	0.7 ± 0.3	9 ± 4
	7,8,9	0.6 ± 0.2	10 ± 5

In all experiments, the AIS converged substantially faster by at least an order of magnitude. It can also be remarked that some datasets were more difficult to train than others which appears to be consistent in the two training strategies. For instance, the sonar dataset required typically more iterations.

**Fig 10:**
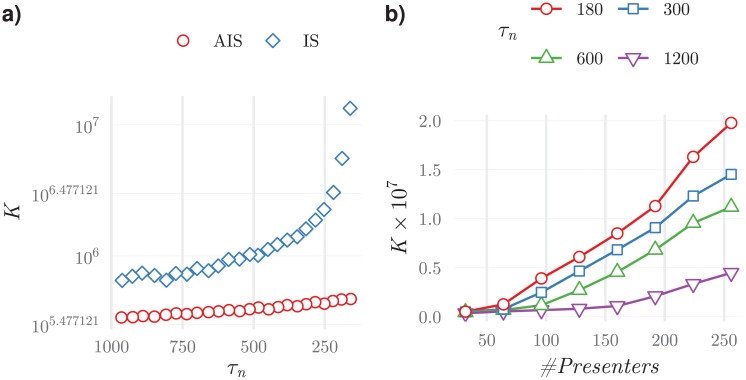
Average number of iterations, K, as a function of a) the pairing duration threshold *τ*_*n*_ reached, and b) the number of presenters in the population (which is equal to the number of detectors) for different values of the *τ*_*n*_ reached and for simulations with connectivity, *C* = 12. The results on the left, show that the new strategy requires a considerable lower number of iterations to reach configurations with the same *τ*_*n*_, than the more immunologically plausible alternative. This is especially meaningful when *τ*_*n*_ < 250. Results on the right show that for systems with fixed connectivity, increasing the population size increases the computational time required for training roughly linearly.

In these results the target value of 180 iterations was chosen because it corresponded to a pairing duration that could be attained within an acceptable computational time (typically, no more than 15 minutes) by both training strategies. To complement these results, in Fig 10a) the number of iterations required to have all agents pairing durations below *τ*_*n*_ is plotted. These results are an indirect measure of the IList organisation, i.e., of the number of educated positions as analysed in Fig 9. Results in Fig 10a) considered populations trained with the wine dataset, when the normal training data had wines with quality scores between 5 and 7. These results represent the typical behaviour of *K*, also observed in the other systems.

These results show that the immunological strategy requires a number of iterations that grows faster (faster than exponentially) than the artificial immune strategy for an equivalent level of IList organisation. Therefore, these results agree qualitatively with those described by the simplified model for the education of a single IList.

Results in Fig 10b) show that it is possible to increase the number of features displayed by presenters increasing only linearly with *N*_*f*_ the computational time. This is true provided the connectivity is kept constant.

#### Anomaly detection performance

**Fig 11:**
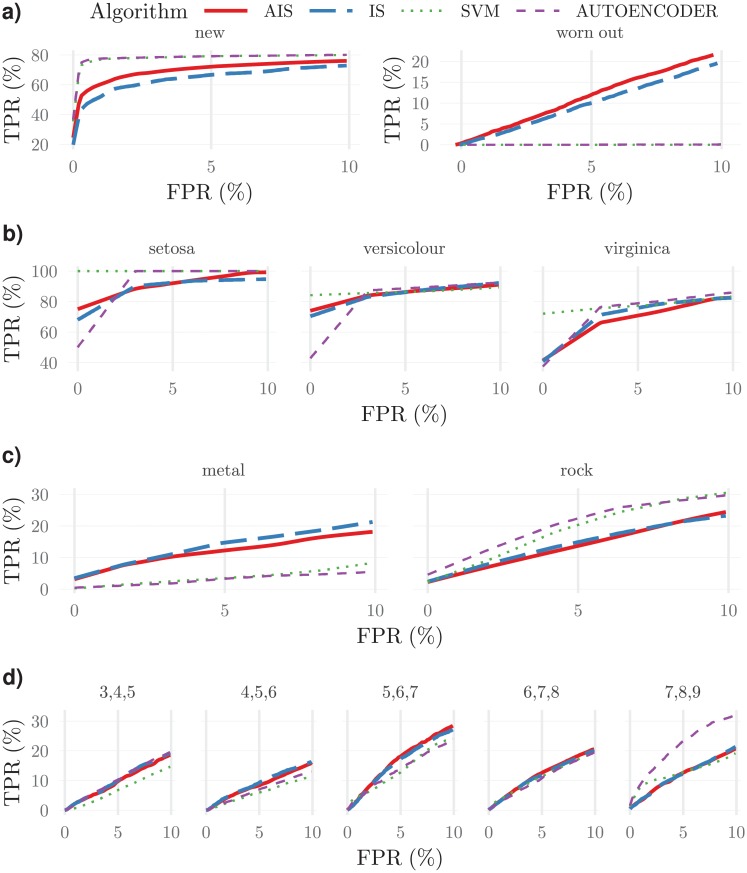
Average ROC curves obtained from 10 fold Monte-Carlo cross-validation, for the several datasets and for the several normal class sub-sets defined in Table 1 and mentioned in each plot header. Shown are results from the two cellular frustrated algorithms (CFAs) (parameters as in Table 3) and the one-class SVM with the kernel producing the best results. The quantitative value of the true positive detection rate (TPR) at at a 10% false positive rate (FPR) as well as its standard deviation can be found in S1 Table. Overall these results show that cellular frustrated algorithms achieve comparable, if not better precisions than one class SVMs.

To compare the precision of the new training strategy with the immunologically more plausible strategy, ROC curves for anomaly detection tests with the several datasets were obtained (Fig 11). Furthermore, a comparison with the one-class support vector machines and autoencoders is also provided.

To establish a fair comparison among the several methods, all algorithms used the same samples for training and testing. Furthermore, since the aim is also to evaluate the robustness of different algorithms, the normal class used for training was chosen by selecting sub-sets with the different classes in each dataset (see Table 1). All the results presented next used a same fixed set of parameters and, in the case of the SVM, the same kernel.

The set of results in Fig 11 allow drawing two main conclusions by analysing the *TPR* at a 10% *FPR* on the several plots. First, the artificial intelligence algorithm proposed here has similar precision to the more immunological plausible alternative. Therefore, the new algorithm is interesting especially because it increases training speed by one-fold, at least.

The other important result is the comparison with the results obtained from one-class SVMs and deep autoencoders. These two algorithms produce very similar results, differing only appreciably in two tests in the wines datasets: tests 8 and 12 (first and last plots in Fig 11d). In comparison with CFAs, these methods are, in some cases, more precise—for instance, in the ball bearings dataset, when the normal class consists of samples obtained from new ball bearings, or, in the sonar dataset, when the normal class consists of data arising from sonar signals reflected by rock. However, on both cases, when the normal class is formed by samples from the other class, detection is not achieved at all. This suggests that at least, CFSs present more robust results. Of course that it may be argued that SVM methods require a judicious choice of the kernel in each case. This however, is problematic in many applications especially when semi-supervised anomaly detection is required. In the S2 Fig results obtained with other kernels are also presented, demonstrating overall poorer performances.

It should be mentioned that the ROC curves presented in Fig 11 result from a 10 fold Monte-Carlo cross-validation. Variability in these experiments exists but it is fairly similar among the two cellular frustrated algorithms, as can be appreciated in S1 Table. SVMs have smaller variabilities, which can be expected given the stochastic nature of CFAs.

#### Anomaly detection robustness

The particular choice of parameters can be critical and therefore, needs to be discussed to evaluate the robustness of these results. This discussion is not always easy to make with total fairness since, in semi-supervised anomaly detection only information from a single class is available. As a result, for any new method the developer tests countless variations and incorporates his knowledge in selecting standard parameters. Certainly, only with a growing number of studies and on different datasets will it be possible to establish definite conclusions on how different algorithms compare.

The parameters used in CFSs were relatively simple to establish and are listed in Table 3. This seems a long list, however results do not critically depend on most of them. In many cases, their choice follows naturally from the detection mechanisms identified in [3, 43].

**Table 3:** Parameters used in cellular frustration algorithms.

Threshold Probability *v*_*max*_(%)	5
Number of educated populations included in the repertoire, *N*_*pop*_	12
Detectors connectivity, *C*	20
Education Window *W*_*τ*_ (iterations)	10^4^
Education time sampling window *T*_*S*_ (iterations)	100
Detection window *W*_*d*_ (iterations)	10^4^
Anergy time *τ*_*A*_ (iterations)	5
Detection pairing duration to activate response *τ*_*act*_ (iterations)	*τ*_*A*_
Calibration parameter *f*	0.1

For instance, the threshold probability *v*_*max*_ should be small but nonzero, to allow discrimination of outliers and of abnormal samples. Of course that the best value for *v*_*max*_ should depend on the dataset because, if only outliers are to be found then *v*_*max*_ should be zero. On the other side, if no outliers exist, then detectors using *v*_*max*_ = 0 only participate in frustrating the dynamics. In the supplementary materials 2 (S3 Fig) the average *TPR* obtained with a *FPR* of 10% is shown for the different datasets and for *v*_*max*_ = 0, 5, 10%. These results show that detection can vary with *v*_*max*_. This is clear in the results obtained with the ball bearings and the wines datasets. From these results it seems clear that *v*_*max*_ = 5% is generally a good compromise.

In [3] it was shown that a repertoire composed of several independently educated sets of detectors, could improve detection rates, when a single outlier was presented. This happens because the number of ILists that can rank outliers on top positions is increased. The results shown in S1 Table capture some improvement when the number of educated populations included in the repertoire increases from 1 to 12. However, the Number of educated populations included in the repertoire does not seem to have a critical impact in anomaly detection rates. This conclusion is valid as far as the current datasets are concerned. It is always possible that in other datasets the most frequent anomaly would correspond to the appearance of a single outlier. Then, the influence of the number of populations in the results could be important [3]. This can be particularly relevant in the context of intrusion detection, because attackers try to explore such vulnerabilities. In the remaining results presented next we choose repertoires with 12 populations.

In [3, 43] it was shown that detector’s connectivity—the number of presenters a detector can interact with—could have an impact in anomaly detection performances and also on training convergence. To understand this it should be recalled that, using the plausible immunological training strategy, only a few top positions (typically not larger than 10; see Results in section) will be ordered. That is, on top positions in ILists there will be mostly signals delivered by presenters of the opposite subtype. In the following positions, ILists are relatively disordered, with signals delivered by presenters of both subtypes. As a result, in populations with large connectivities, the probability that a detector interacts with signals in the ordered region, is small. Consequently detection performances tend to be poorer. Furthermore, training also requires more time to reduce *τ*_*n*_. On the opposite extreme, for very small connectivities, fluctuations in the number of signals present in a sample and ranked on ILists top positions increase. This also leads to a less organized dynamics.

Two types of results confirm these analyses. First in S4 Fig it is shown that, for the immunological strategy the number of iterations required to reach a given maximal pairing duration *τ*_*n*_, has a minimal value for intermediate connectivities. Interestingly convergence of the artificial intelligence algorithm became much more insensitive to connectivity changes. In what concerns the impact of connectivity on the anomaly detection accuracies, results are much less clear for both strategies, and this is likely to be due to the relatively small number of independent features present in the datasets used. However, in the immunological plausible strategy there are datasets—for instance, the ball bearings dataset—in which the largest connectivities can produce clearly poorer results. In some cases, however, results are not very sensible to changes in connectivity, as happens for instance, with the sonar dataset. In any case, and interestingly, anomaly detection performances of the new training strategy are almost insensitive to connectivity changes for the studied datasets (see S5 Fig) except if connectivity is extremely small. This, we believe, is due to the improved ordering in ILists. This result is important because it reduces the number of parameters to tune. Therefore, as a general conclusion, the connectivity should be chosen to take moderate values, within the range of a few dozens, specially for computational convenience reasons.

In order to gain good generalization capabilities, it was shown that the time sampling window *T*_*S*_ should be small [43] and the education window *W*_*τ*_, used to decrease *τ*_*n*_, should be large—i.e., *W*_*τ*_/*T*_*S*_ ∼ 100—to correct detectors only depending on their performances in a large number of samples. The results we obtained (see S6 Fig) do not exhibit such a dramatic effect as the one reported previously [43]. In some cases, can even seem to contradict these previous results (as in the iris dataset, with virginica as normal class, or in the sonar dataset with metal as normal class), although we believe that this can be due to the small number of samples in these examples. More interesting, is the robustness demonstrated by the new AIS strategy to variations in *W*_*τ*_ /*T*_*S*_. This is interesting because again it shows that results became independent of the choice of these parameters.

Next, with respect to the detection window *W*_*d*_, this was chosen to be 10^4^ because one needs good statistics to establish pairing lifetimes. However, as can be appreciated in S7 Fig, increasing this value further does not further improve results.

The anergy time *τ*_*A*_ was chosen having in mind that the distribution of pairing durations decays exponentially. Therefore the occurrence of pairings lastings longer than typical pairing lifetimes may not provide additional information. On the contrary, using small values for *τ*_*A*_ improves statistical accuracy since more pairings can be tested. In fact, since detectors minimum pairing lifetime is of the order of 5, the number of pairings lasting longer than this value can represent 40% of the total number of pairings. Therefore, *τ*_*A*_ ≃ 5—the value used in [43], seems an acceptable choice. However, values up to *τ*_*A*_ ≃ 20 would produce similar, if not slightly better results (see S8 Fig). Finally, we should mention that *τ*_*A*_ should be always larger or equal to 2 because otherwise generalized kinetic proofreading would not take place. However, we should note that in a single iteration there are agents that are selected by more than 10 agents for interaction. Therefore, even for small *τ*_*A*_ values, kinetic proofreading is already deeply present.

Finally, the calibration parameter *f* was chosen to be 0.1. However, its impact in the anomaly detection performance of the algorithm is also reduced provided *f* is not too small (see S9 Fig). The *f* parameter was first introduced in [58] to take into account knowledge of the typical pairing durations observed in the calibration stage. Since detection mechanisms involve the number of long lived pairings, it could be expected that only those agents performing the longest pairings should be considered. The results we present in S9 Fig, show that if *f* < 0.05, performances deteriorate. This can be due to the fact that not enough agents that play an important role in the discrimination are participating. Therefore, *f* should take larger values.

The interesting result if that if *f* takes maximum values the results are almost not changed. This suggests that the calibration stage could be eliminated, which represents an important simplification in the algorithm. However, it is not clear to us how general this conclusion may be, especially having in mind future developments of the algorithm. This was the reason why the calibration stage was kept in this work. To conclude, while there are several parameters at play, whose values have to be defined, selection of reasonable values is not difficult to establish following our understanding of the detection mechanisms. Consequently, the results presented in Fig 11 are robust relatively to their variation.

In contrast, the choice of the kernel in one-class SVM influences considerably the results. For the results presented in Fig 11, we chose the kernel that gave better overall results (a polynomial kernel with degree 2 and *c* = 0, *ν* = 1/*N*_*f*_ [56]). A comparison with results obtained with other kernels can be found in S1 Fig.

#### Anomaly detection mechanisms

Detection in CFAs can arise from two types of mechanisms: detection of outliers or detection of an increased number of absent frequently displayed signals. The two mechanisms can take place simultaneously, and consequently except in special cases (as those discussed in [43]), it is not always easy to clearly point which mechanism is playing a crucial role. In order to enlighten this point with respect to the present datasets, Table 4 compares the performance of CFAs with *v*_*max*_ = 0% and *v*_*max*_ = 5% and with results deriving from two methods based on simple rules. These two methods simply count the number of rare signals appearing in each sample in the detection stage and establish the TPR as the fraction of anomalous samples having a number of rare signals larger than found in 90% of the normal samples.

**Table 4:** *TPR* for 10% *FPR* for the two strategies (AIS and IS) when *v*_*max*_ = 0% and *v*_*max*_ = 5%. On the last three columns are the results using a simple rule using simply the number of rare signals mapped from each sample, as explained in the text.

test	dataset	normal training data	AIS		IS		#rare signals		#rare sig. in ILists
			0%	[0, 5]%	0%	[0, 5]%	0%	5%	[0, 5]%
1	ball bearings	new	79.8	76.1	79.8	74.5	80.6	79.6	79.6
2		worn out	10.4	22.0	10.4	19.7	8.0	12.1	13.4
3	iris	setosa	98.8	99.5	95.6	96.4	99.4	99.4	100.0
4		versicolour	90.9	89.8	90.1	92.5	90.6	90.6	90.3
5		virginica	82.5	82.3	84.4	81.5	74.5	74.5	78.4
6	sonar	metal	19.5	17.4	17.7	20.9	10.3	12.4	19.3
7		rock	22.3	25.9	23.2	23.4	29.3	29.2	26.9
8	wines	3,4,5	12.4	18.5	12.5	19.6	13.8	14.2	17.6
9		4,5,6	11.8	16.5	11.8	16.3	12.4	13.2	15.6
10		5,6,7	15.8	28.2	15.8	26.9	16.7	26.1	28.1
11		6,7,8	13.4	20.7	13.4	20.1	14.2	20.0	20.1
12		7,8,9	18.7	20.7	18.7	20.9	21.2	17.0	21.1

The two methods based on simple rules differ on how sample elements (i.e., features) are mapped onto rare signals. In the first method (columns 8 and 9 in Table 4) an element in a sample is mapped onto a rare signal if it lies in a tail (either, left or right tail) of the corresponding feature distribution. Only data used during training is used to estimate the tail region. Therefore, for 0% tails (column 8 in Table 4), only sample features outside the range of values observed during training produce rare signals. The second method (results in the last column in Table 4) counts the number of rare signals in the detectors ILists used in the CFAs with results listed in columns 5 and 7 of Table 4).

Analysing Table 4 it is possible to conclude that:

1. in some tests, detection of outliers is responsible for the anomaly detection. This happens in tests 1, 3, 4, 7, 12, for which the simple rule counting the number of outliers in samples (the number of rare signals in 0% tails) produces similar TPRs than CFAs with *v*_*max*_ = 0%.
2. detection in tests 6, 10, 11, 12 can be explained as resulting from the presence of a larger number of features with values in the tails than typically happens in normal samples, since the number of rare signals in ILists is enough to explain CFAs results with *v*_*max*_ = 5%. Still, it should be noted that tails of different sizes must be considered and it would not be enough to consider a single tail with 5% of the values. Therefore, even if a simple rule could be devised, it requires already some computational complexity.
3. test 2, and to a lesser extent, tests 5 and 8, indicate detection of correlations in the absence of frequent signals.

In general terms, one can conclude that, although the majority of datasets may not require algorithms as elaborate as CFAs to achieve results with the accuracies reported here, it is clear that this cannot be known in advance, and also that some tests demonstrate the need for using this type of algorithms. Indeed, test number 2 clearly demonstrates that this class of algorithms is needed to perform accurate anomaly detection.

## Conclusions

The cellular frustration framework showed a new way of looking into cellular interactions in the adaptive immune system and how they could work to produce an effective surveillance system. In particular, in a recent work we showed that cellular frustrated systems could be used to perform location statistical tests with performances that could outperform well known statistical tests, like the t-test or the KS-test [43]. In that work, using synthetic data we also showed that CFSs could compete with support vector machines.

The goal of this work was two folded. On one side we wanted to test cellular frustration algorithms using real datasets. On the other side we wanted to understand if simpler versions of the cellular frustration algorithm could be devised to produce similar, if not better results. These improved algorithms would not have to follow the immunological reality closely, taking instead a more general artificial intelligence approach. Therefore, in this work, in the training stage, instead of replacing detectors establishing the most stable pairings by new agents, small corrections were introduced in their ILists to incorporate this new knowledge. The new algorithm proved to be at least one-fold more efficient in computational terms, and anomaly detection rates remained equivalent to the ones obtained with the more immunological version of the algorithm. Furthermore, the new algorithm also gained robustness, since it was found that anomaly detection rates only depended on a single parameter (within the reasonable ranges of variation of the parameters). This robustness improvement can also increase by one extra fold the computational efficiency of the algorithm since it reduces the size of the detectors repertoire used.

Therefore, the algorithm proposed here reduced the complexity in initial proposals [3, 43, 58] by eliminating the need of using the calibration stage and by reducing the number of parameters that one should tune to only one. It should be mentioned, however, that these conclusions are restricted to semi-supervised anomaly detection applications with stationary data. It is possible that in dynamic contexts or in the adaptation of the algorithm to classification tasks, some of these conclusions do not apply.

In this work we also compared CFAs with SVMs and deep autoencoders (DAEs). SVMs and DAEs showed similar accuracy performances. In comparison with CFAs it was found that CFAs displayed more consistent results because, in several cases SVMs and DAEs were unable to identify anomalies—this did not happen with CFAs. Robustness can be critical for general semi-supervised anomaly detection applications because then, little is known about the type of anomalies that will appear. On the other side, it should be mentioned that SVMs and DAEs have the advantage of being considerably faster than CFAs (by almost two orders of magnitude) when datasets have a small number of samples and a small number of features. For large datasets CFAs can be competitive, although we leave investigation on this issue for future work.

To sum up, this work highlighted how frustration can be used to generate another type of swarm behaviour with practical relevance. Here we showed that CFAs can be competent data mining algorithms for anomaly detection tasks and that several different implementation strategies can be developed, contributing and receiving inspiration from research in theoretical immunology and the artificial intelligence field.

## Supporting information

S1 Fig Variation of the maximum pairing duration with the number of presenters. (PDF)

S2 Fig Impact on results of different SVM kernel choices. (PDF)

S3 Fig Impact on results of variations of *v*_*max*_. (PDF)

S4 Fig Impact of varying connectivity *C* on *τ*_*n*_. (PDF)

S5 Fig Impact on results of varying detector’s connectivity. (PDF)

S6 Fig Impact on results of varying *T*_*S*_. (PDF)

S7 Fig Impact on results of varying *W*_*d*_. (PDF)

S8 Fig Impact on results of varying *τ*_*A*_. (PDF)

S9 Fig Impact on results of varying *f*_*c*_. (PDF)

S1 Table Average *TPR* obtained for the several algorithms. (PDF)
