# Supplementary material for: Cellular frustration algorithms for anomaly detection applications
Source: PLoS One. 2019 Jul 8;14(7):e0218930. doi: 10.1371/journal.pone.0218930 (PMC6613704; doi:10.1371/journal.pone.0218930)
Supplement: S1 Fig — (PDF) [file pone.0218930.s001.pdf]

S1 Fig. - Variation of the maximum pairing duration with the number of presenters

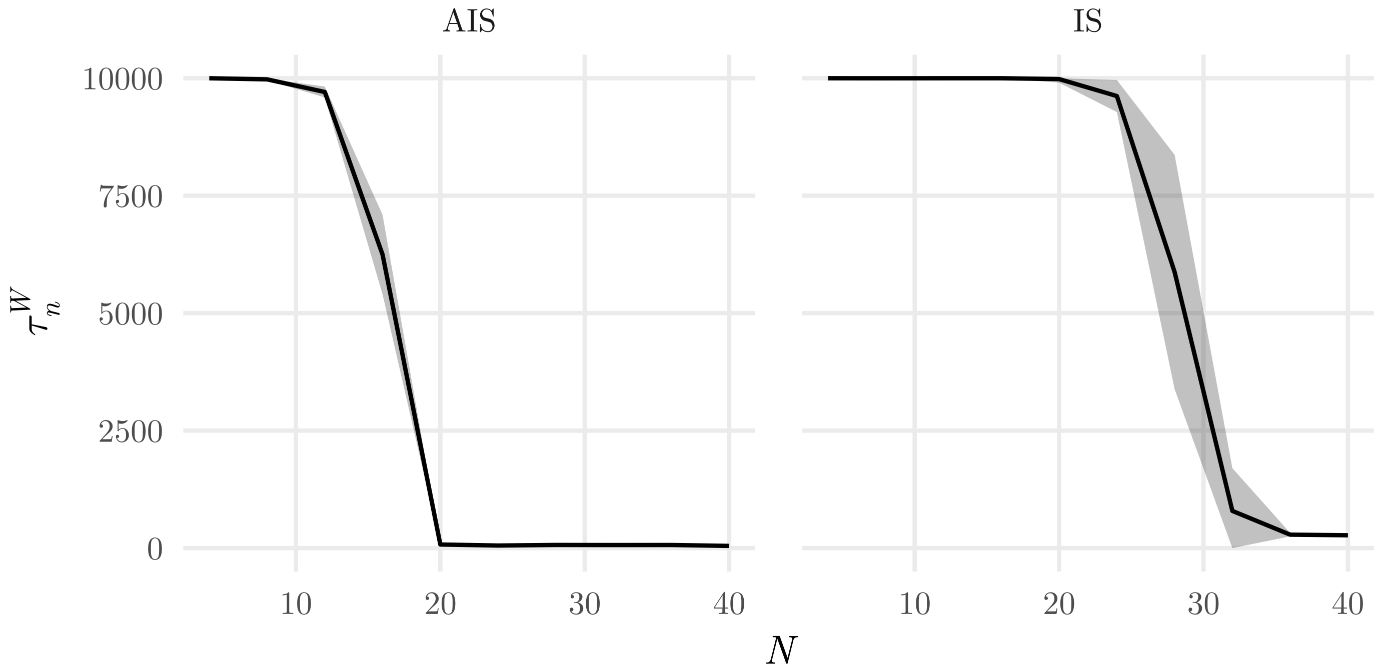

Figure 1: Maximum pairing durations, τ_n^W, as a function of the number of presenters N during testing. When the number of agents is too small, the system can be blocked in a stable configuration or, at least, some pairs may form blocking pairs. This blocked dynamics does not occur for large systems because there will always be unpaired agents that can destabilize paired agents. For this reason, for datasets with a small number of features, as in the case of the iris dataset, sample features were replicated an even number of times to increase the number of presenters in the population. This figure displays typical results obtained after training 12 populations on a single sample of the Iris dataset for $W_t = 4010^6$ iterations, and when the number of presenters varied between 4 and 40 in multiples of 4. The solid line represents the average maximum pairing duration for the 12 different trained populations run for $W = 10000$ iterations. In grey it is accounted the standard deviation for these results. These results show that populations can have blocking pairs when the number of presenters is smaller than 32. For this reason, in our studies we decided to replicate the number of presenters until reaching 32.
