# Supplementary material for: Cellular frustration algorithms for anomaly detection applications
Source: PLoS One. 2019 Jul 8;14(7):e0218930. doi: 10.1371/journal.pone.0218930 (PMC6613704; doi:10.1371/journal.pone.0218930)
Supplement: S2 Fig — (PDF) [file pone.0218930.s002.pdf]

S1 Fig. - Impact on results of different SVM kernel choices

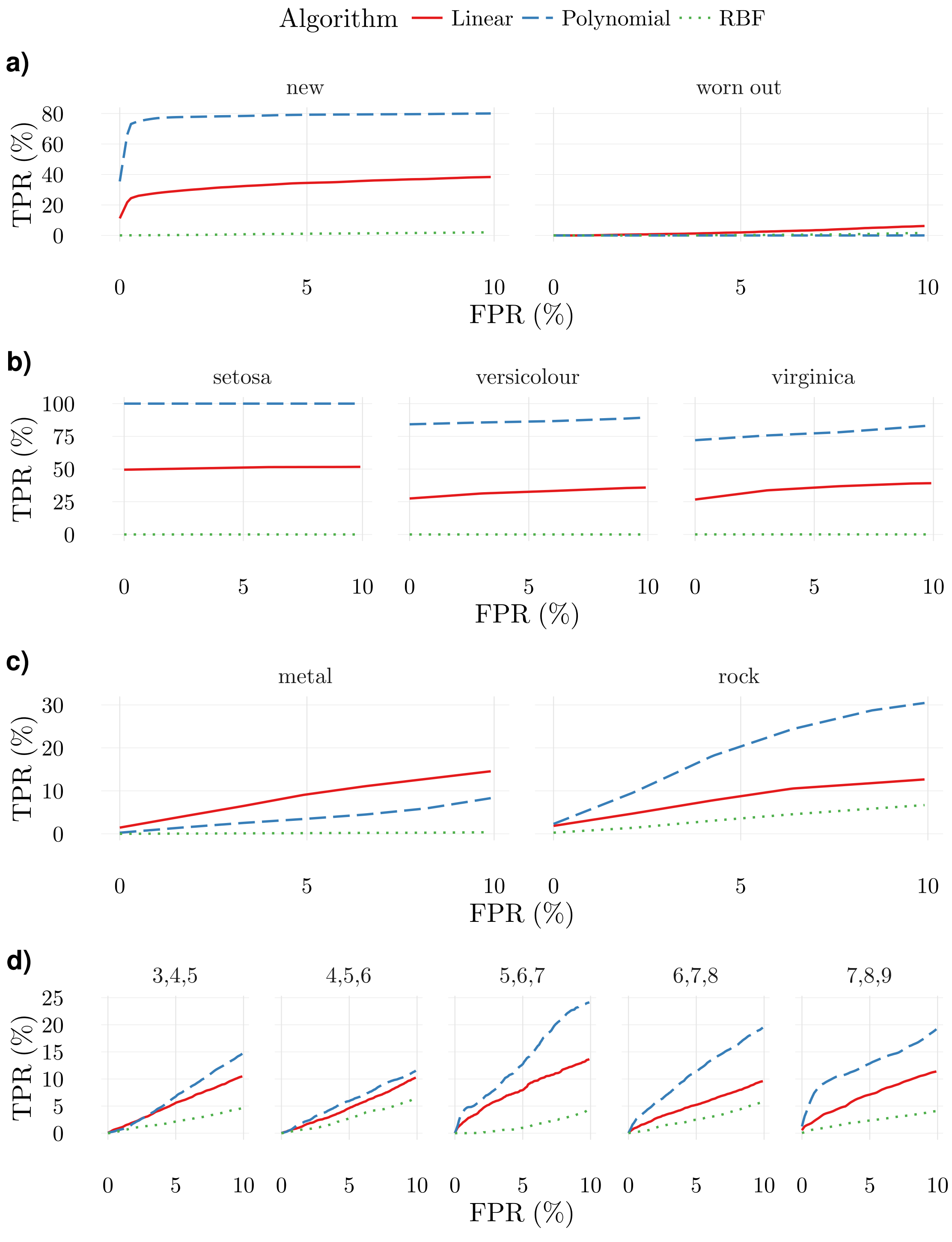

Figure 1: Impact of the kernel choice on the average TPR for the different datasets considered. Overall discrimination is best when the polynomial kernel with degree 2 is used. This was the kernel used in the results reported in this article. In these results, the γ parameter used for the Gaussian kernel was $\gamma = 1/\text{dataset dimension}$, while the polynomial and linear kernels took $c = 0$. All the results were obtained training models with $v = 1/N_f$. These are the default parameters considered in [36].
