# Supplementary material for: Cellular frustration algorithms for anomaly detection applications
Source: PLoS One. 2019 Jul 8;14(7):e0218930. doi: 10.1371/journal.pone.0218930 (PMC6613704; doi:10.1371/journal.pone.0218930)
Supplement: S3 Fig — (PDF) [file pone.0218930.s003.pdf]

S2 Fig. -Impact on results of variations of v_{max}

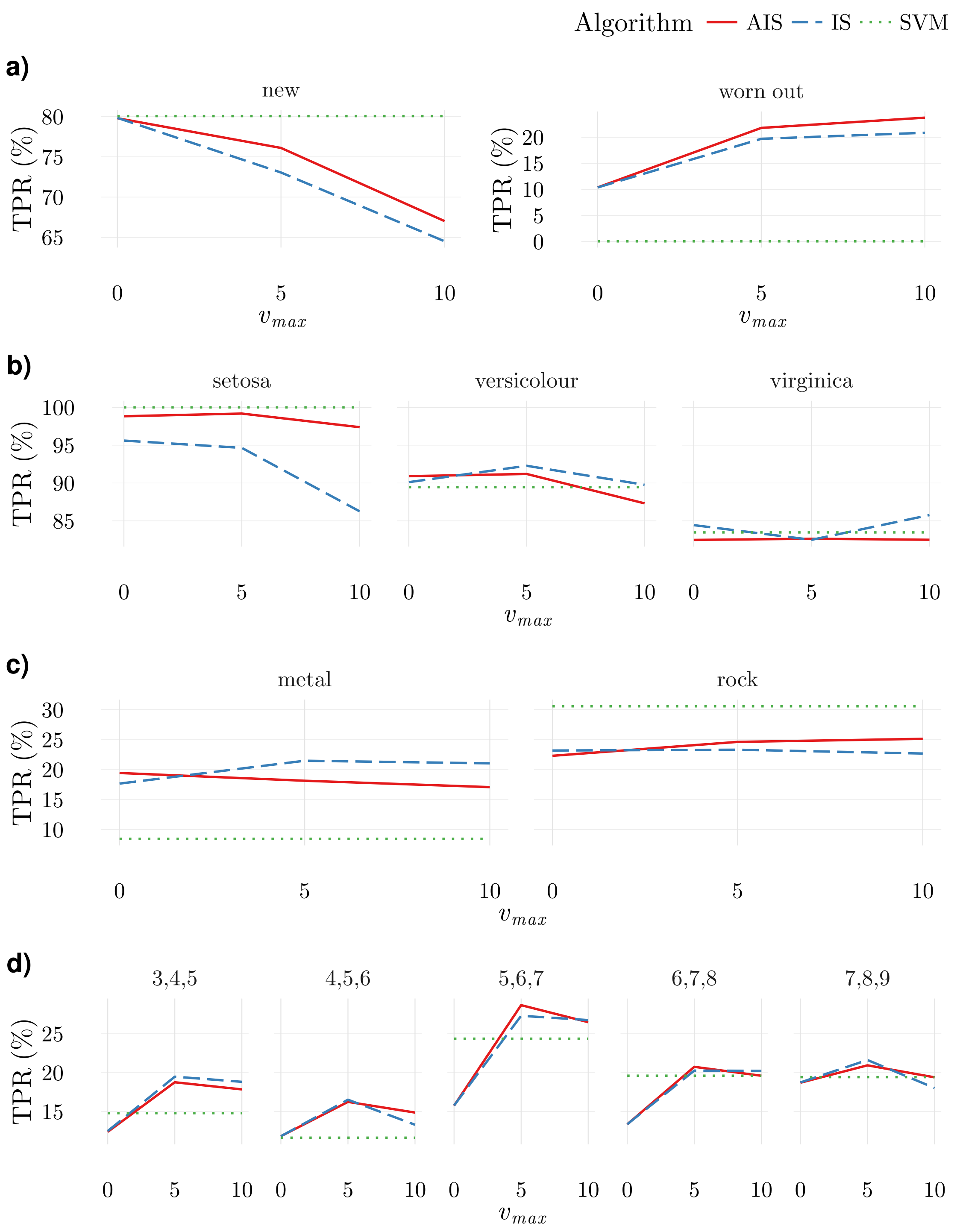

Figure 1: Average true positive rates (TPR) obtained when the maximal probability of perceiving the information displayed by a presenter as a rare signal is v_{max} for the different strategies (AIS and IS) and on the different datasets. By order of appearance, a), b), c), d), describe the results for the ball bearings, iris, sonar and wine quality datasets, respectively. The title of each plot represents the dataset class used as the normal class, while the remaining samples were considered as abnormal. All results were obtained after 10 executions, each with an initial different set of samples. Results refer to a 10% FPR. Overall discrimination is best for $v_{max} > 0$ which means that discrimination is not exclusively due to the presence of outliers.
