# Supplementary material for: Cellular frustration algorithms for anomaly detection applications
Source: PLoS One. 2019 Jul 8;14(7):e0218930. doi: 10.1371/journal.pone.0218930 (PMC6613704; doi:10.1371/journal.pone.0218930)
Supplement: S4 Fig — (PDF) [file pone.0218930.s004.pdf]

# S4 Fig. - Impact of varying connectivity $C$ on $\tau_n$

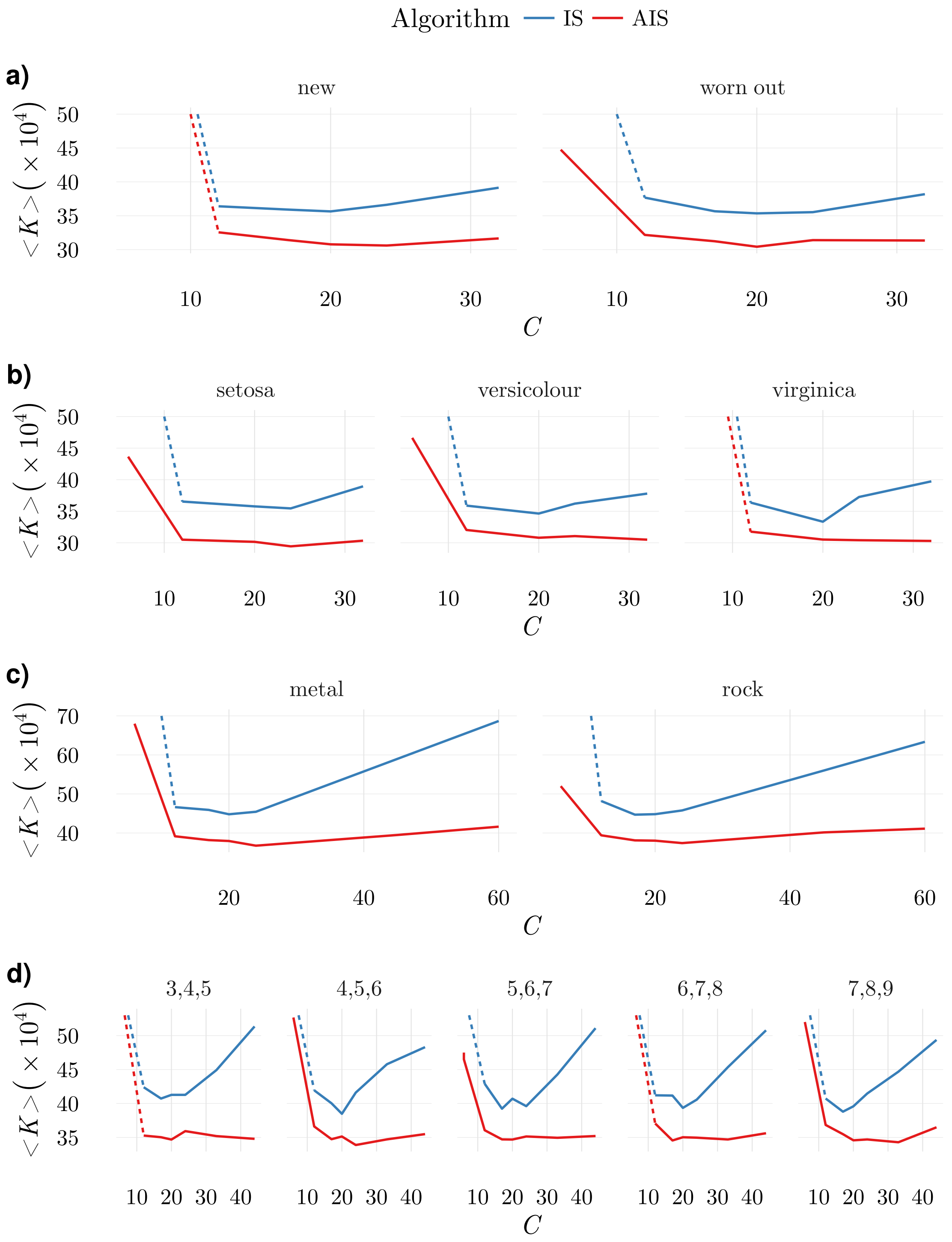


Figure 1: Average number of iterations required to reach the fixed maximal pairing duration $\tau_n = 560$ during education, for 10 different populations. These results show that for the immunologically plausible algorithm, smaller connectivities can considerably improve the convergence speed. This effect is almost absent in the new artificial intelligence algorithm, which highlights the good convergence behaviour of the new algorithm even when large connectivities are considered. When the connectivity is too small - the smallest connectivity considered was $C = 6$ in all cases - convergence slows considerably, and in some cases repertoire education did not converge within the maximal number of iterations attended ($20 \times 10^6$ iterations). In the later cases, dashed lines are used to serve as guides to the eye, to illustrate a steep increase in the number of iterations required for convergence.
