# Supplementary material for: Cellular frustration algorithms for anomaly detection applications
Source: PLoS One. 2019 Jul 8;14(7):e0218930. doi: 10.1371/journal.pone.0218930 (PMC6613704; doi:10.1371/journal.pone.0218930)
Supplement: S5 Fig — (PDF) [file pone.0218930.s005.pdf]

# S5 Fig. - Impact on results of varying detector's connectivity

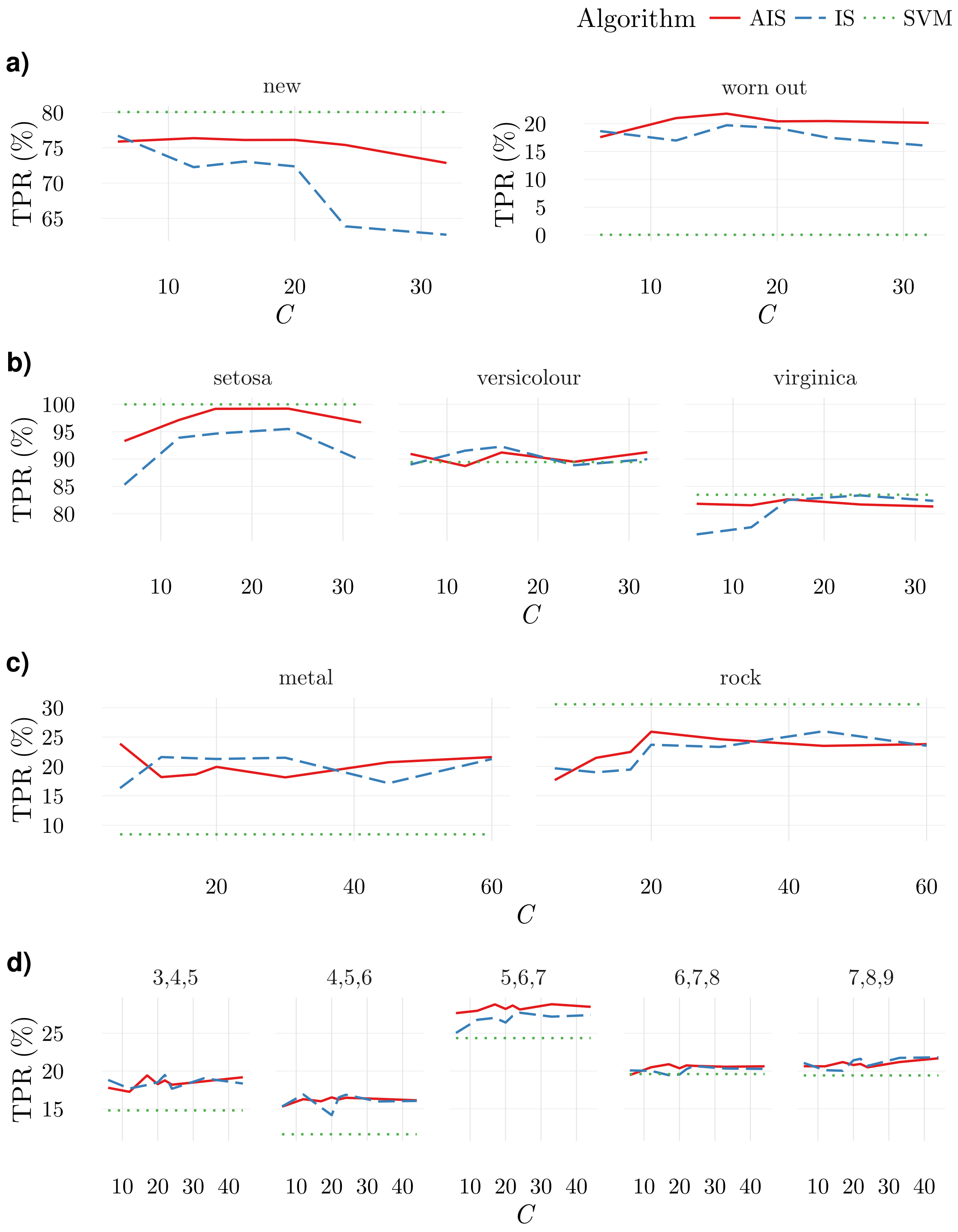


Figure 1: Average $TPR$ obtained for different values of detectors connectivity, $C$. Overall increasing detectors connectivity should decrease discrimination capability. This is true for the IS and for systems with a large number of features which are also harder to educate. For the datasets analysed here the impact of $C$ is only mild since the number of features in the several cases is relatively small. In any case, it seems clear that $C$ should not be too small (in this case education can be very hard; see SM3) and there can be an advantage in not having full connectivity.
