# Supplementary material for: Cellular frustration algorithms for anomaly detection applications
Source: PLoS One. 2019 Jul 8;14(7):e0218930. doi: 10.1371/journal.pone.0218930 (PMC6613704; doi:10.1371/journal.pone.0218930)
Supplement: S6 Fig — (PDF) [file pone.0218930.s006.pdf]

S6 Fig. - Impact on results of varying T_S

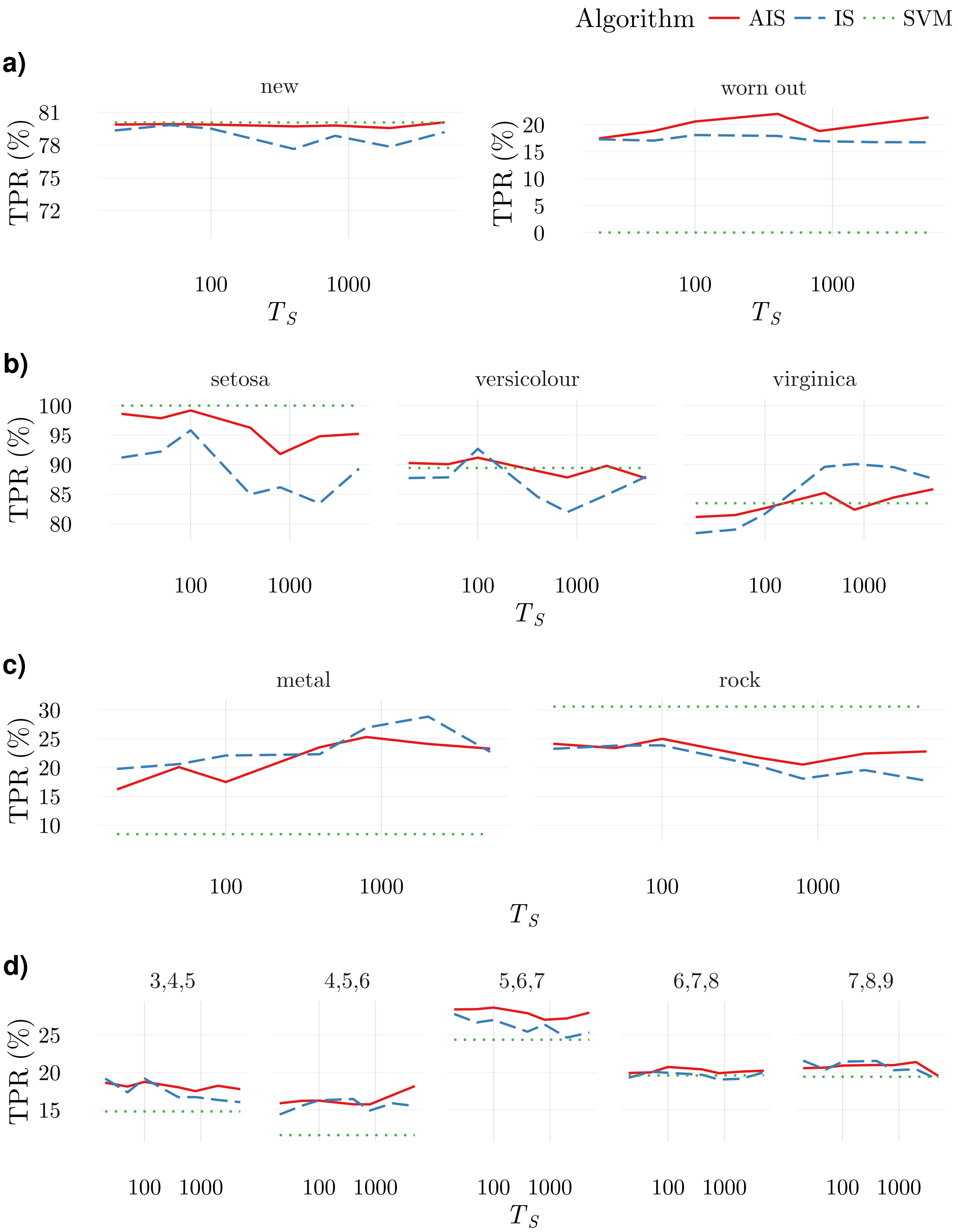

Figure 1: Impact of changing T_S, the number of iterations separating the presentation of different samples during training, when the time interval W_τ required to decrease the pairing duration threshold, τ_n, is held fixed. These results show that in the AIS the impact of T_S in results in extremely reduced. This is due to the fact that education in the AIS has memory making it less critical to present a large number of samples in a short time interval. Therefore, for the new strategy, the impact of T_S variations is small. The values of T_S used in this plots is: $T_S = 20, 50, 100, 400, 800, 2000, 5000$. In each point,an average over 10 different tests involving different normal samples, have been calculated.
