# Supplementary material for: Cellular frustration algorithms for anomaly detection applications
Source: PLoS One. 2019 Jul 8;14(7):e0218930. doi: 10.1371/journal.pone.0218930 (PMC6613704; doi:10.1371/journal.pone.0218930)
Supplement: S7 Fig — (PDF) [file pone.0218930.s007.pdf]

S7 Fig. - Impact on results of varying W_d

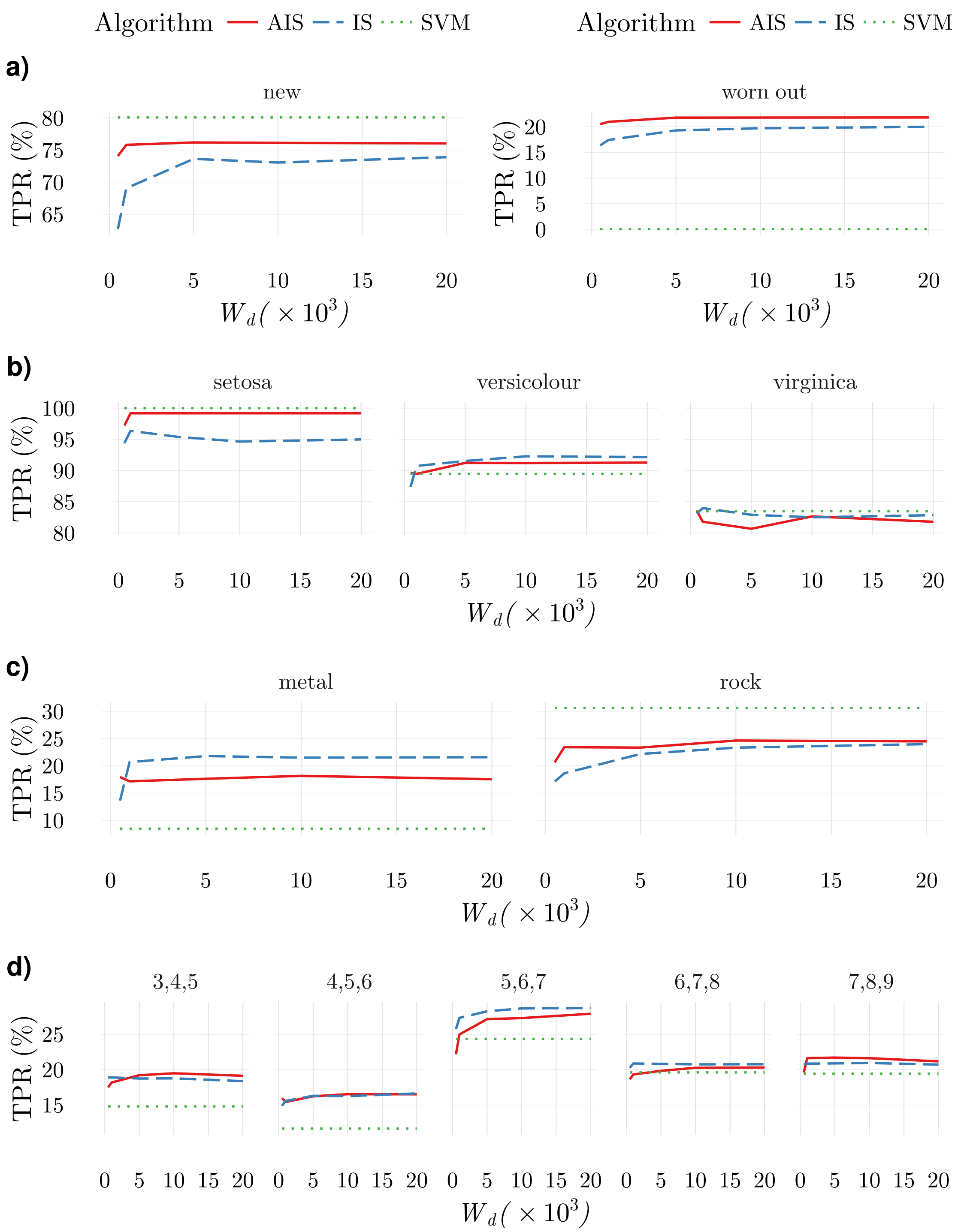

Figure 1: Average $TPRs$ obtained for different values of W_d. Overall these results show that beyond $W_d \sim 5000$ iterations, the performance of the method becomes stable. Therefore, W_d is also a parameter requiring no tuning. In the results reported in this paper we used $W_d = 10000$.
