# Supplementary material for: Cellular frustration algorithms for anomaly detection applications
Source: PLoS One. 2019 Jul 8;14(7):e0218930. doi: 10.1371/journal.pone.0218930 (PMC6613704; doi:10.1371/journal.pone.0218930)
Supplement: S8 Fig — (PDF) [file pone.0218930.s008.pdf]

S8 Fig. - Impact on results of varying τ_A

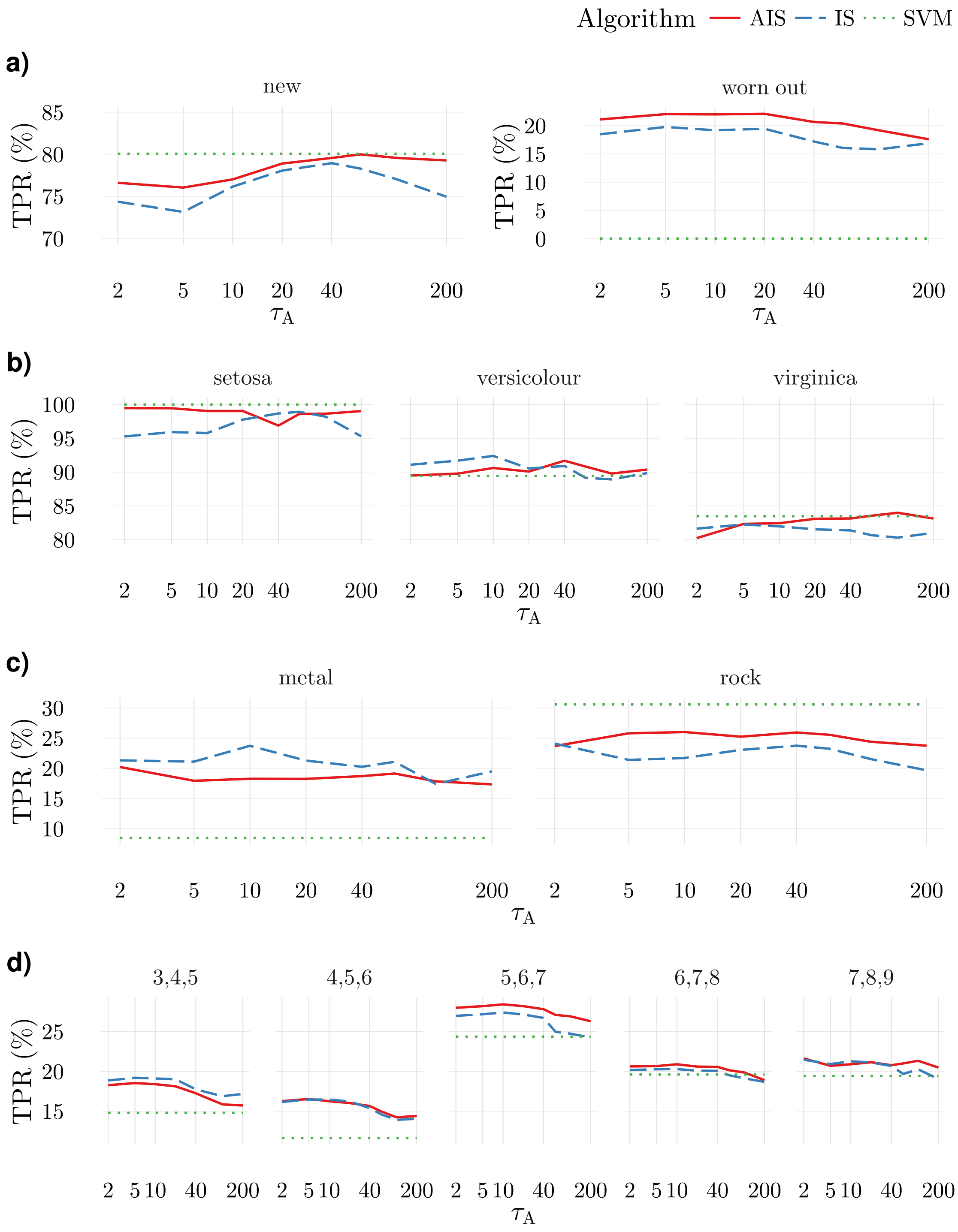

Figure 1: Average true positive rates obtained for different values of τ_A (and consequently τ_{act}, since $\tau_{act} = \tau_A$). By order of appearance, a), b), c), d), describe the results for the ball bearings, iris, sonar and wine quality datasets, respectively. The title of each plot represents the dataset class used as the normal class, while the remaining were considered as abnormal. All results were obtained considering a 10% false positive rate. Overall discrimination improves for $\tau_A >= 5$ which implies that discrimination in these datasets is mostly due to increases of rare ligands.
