# Supplementary material for: Cellular frustration algorithms for anomaly detection applications
Source: PLoS One. 2019 Jul 8;14(7):e0218930. doi: 10.1371/journal.pone.0218930 (PMC6613704; doi:10.1371/journal.pone.0218930)
Supplement: S9 Fig — (PDF) [file pone.0218930.s009.pdf]

# S9 Fig. - Impact on results of varying $f_c$

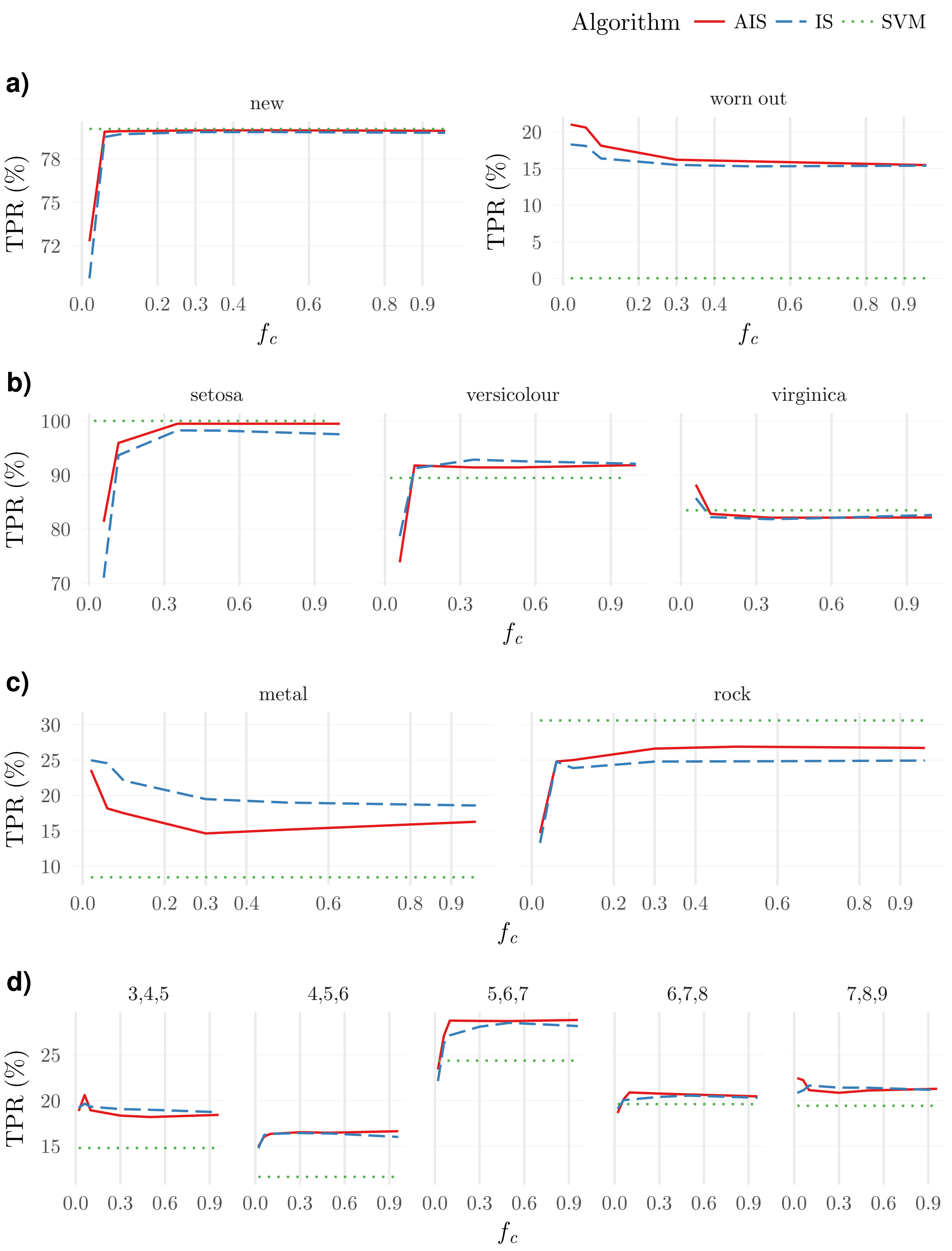


Figure 1: Impact of the variation of $f_c$ on the $TPR$ for a $10\% FPR$. Overall these results show that the maximum value of $f_c$ could be used. This implies that the calibration stage plays almost no role and could therefore be eliminated. This is an interesting improvement in terms of the computational efficiency of the algorithm.
