# Supplementary material for: Cellular frustration algorithms for anomaly detection applications
Source: PLoS One. 2019 Jul 8;14(7):e0218930. doi: 10.1371/journal.pone.0218930 (PMC6613704; doi:10.1371/journal.pone.0218930)
Supplement: S1 Table — (PDF) [file pone.0218930.s010.pdf]

S3 Table-Average TPR obtained for the several algorithms

Table 1: Average TPR and associated standard deviations obtained considering a 10% FPR. Also shown is the impact of increasing the repertoire population size from 1 to 12 populations of detectors in CFAs. Here, $N.D.$ stands for "no detection". Overall, these results show that: 1)larger repertoires improve slightly the accuracy; 2) CFAs have comparable, if not better overall accuracies than one class SVM.

		AIS				IS				SVM	
Number of populations		1		12		1		12			
dataset	normal training data	μ	σ	μ	σ	μ	σ	μ	σ	μ	σ
ball bearings	new	75.8	7.4	76.1	6.3	71.6	9.7	74.5	8.7	80.1	0.2
	worn out	20.1	5.5	22.0	8.1	18.0	3.9	19.7	6.9	$N.D.$	$N.D.$
iris	setosa	94.1	13.6	99.5	1.7	94.2	13.5	96.4	7.2	100.0	0.0
	versicolour	90.0	10.4	89.8	9.9	88.1	13.6	92.5	9.5	89.5	4.5
	virginica	83.6	6.3	82.3	9.5	80.8	11.2	81.5	9.2	83.5	7.5
sonar	metal	17.4	11.6	17.4	9.7	18.1	12.6	20.9	9.3	$N.D$	$N.D$
	rock	24.9	6.8	25.9	5.8	24.3	5.9	23.4	8.0	30.6	3.7
wines	3,4,5	16.5	4.2	18.5	3.4	17.9	4.6	19.6	2.0	14.8	1.5
	4,5,6	15.3	2.9	16.5	2.3	15.9	3.3	16.3	2.4	11.6	1.2
	5,6,7	27.4	1.7	28.2	1.1	25.1	3.2	26.9	2.4	24.4	1.0
	6,7,8	20.2	2.2	20.7	1.5	18.9	2.7	20.1	2.0	19.6	0.8
	7,8,9	19.7	2.2	20.7	3.37	21.6	2.63	21.0	2.4	19.4	1.1
